# Click Chemistry as an Efficient Toolbox for Coupling Sterically Hindered Molecular Systems to Obtain Advanced Materials for Nanomedicine

**DOI:** 10.3390/ijms26010036

**Published:** 2024-12-24

**Authors:** Neyra Citlali Cabrera-Quiñones, Luis José López-Méndez, Carlos Cruz-Hernández, Patricia Guadarrama

**Affiliations:** 1 Materials Research Institute, Universidad Nacional Autónoma de México, Mexico City 04510, Mexico; neyraccq@outlook.com; 2 Biological Systems Deparment, Universidad Autónoma Metropolitana Unidad Xochimilco, Calzada del Hueso 1100, Villa Quietud, Mexico City 04960, Mexico; ljlopez@correo.xoc.uam.mx; 3 Departamento de Ciencias Naturales, Universidad Autónoma Metropolitana Unidad Cuajimalpa, Mexico City 05300, Mexico; carlosacruzhdz@gmail.com

**Keywords:** click chemistry, steric hindrance, polymers, cyclodextrins, fullerenes, nanomedicine

## Abstract

Since its conceptualization, click chemistry in all its variants has proven to be a superior synthesis protocol, compared to conventional methods, for forming new covalent bonds under mild conditions, orthogonally, and with high yields. If a term like reactive resilience could be established, click reactions would be good examples, as they perform better under increasingly challenging conditions. Particularly, highly hindered couplings that perform poorly with conventional chemistry protocols—such as those used to conjugate biomacromolecules (e.g., proteins and aptamers) or multiple drugs onto macromolecular platforms—can be more easily achieved using click chemistry principles, while also promoting high stereoselectivity in the products. In this review, three molecular platforms relevant in the field of nanomedicine are considered: polymers/copolymers, cyclodextrins, and fullerenes, whose functionalization poses a challenge due to steric hindrance, either from the intrinsic bulk behavior (as in polymers) or from the proximity of confined reactive sites, as seen in cyclodextrins and fullerenes. Their functionalization with biologically active groups (drugs or biomolecules), primarily through copper-catalyzed azide–alkyne cycloaddition (CuAAC), strain-promoted azide–alkyne cycloaddition (SPAAC), inverse electron-demand Diels–Alder (IEDDA) and thiol–ene click reactions, has led to the development of increasingly sophisticated systems with enhanced specificity, multifunctionality, bioavailability, delayed clearance, multi-targeting, selective cytotoxicity, and tracking capabilities—all essential in the field of nanomedicine.

## 1. Introduction

In the nanomedicine area, the advancements in molecular-level understanding of signaling pathways and recognition sites associated with various diseases pave the way for more precise therapeutic interventions by designing targeted molecular platforms. However, the synthesis of these platforms demands highly efficient chemical reactions.

Experimental chemistry has extensively progressed, allowing effortless control of the reaction medium, temperature, atmosphere, and even the reaction kinetics by adding a catalyst or avoiding competing functionalities by protecting diverse groups. Nevertheless, the challenge of chemists remains overcoming reactions’ drawbacks like low efficiencies, purity, and selectivity, among others, seeking the simplicity and versatility of synthetic methods to face the challenge of obtaining increasingly sophisticated multifunctional materials for different applications, including medical ones.

In the quest for improvement, and inspired by nature, Sharpless [1] introduced the term “click chemistry” in 2001 to refer to a set of highly efficient and selective reactions, reproducible, and tolerant to different reaction conditions, allowing the obtaining of products by applying simple purification techniques [2].

The most iconic click chemistry reaction is the copper-catalyzed azide–alkyne cycloaddition (CuAAC), which is the catalyzed version of the 1, 3-dipolar Huisgen cycloaddition between azides and alkynes, with inherent inconveniences like high temperatures required, long reaction times (12 to 60 h), and the obtaining of mixtures of products [3]. The addition of copper in the presence of sodium ascorbate, proposed by Sharpless to obtain Cu (I) as a catalyst, resulted in an increased reaction rate of more than 107 times, circumventing the product mixture [4,5].

**Figure 1:**
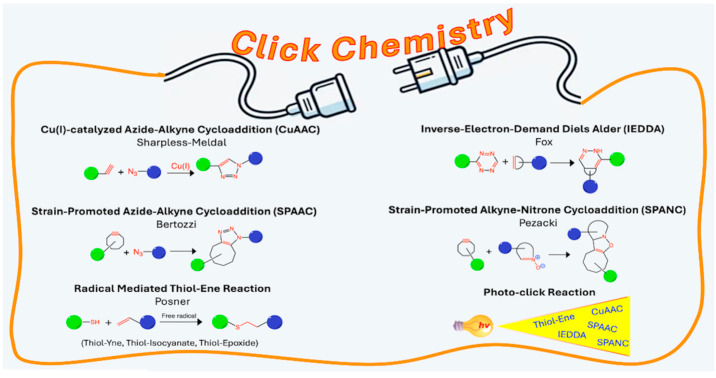
Representatives click reactions. SPAAC stands out for its copper-free nature; SPANC is a click reaction between a nitrone (acyclic or endocyclic) and a diaryl-strained cyclooctyne; IEDDA involves a tetrazine and either a strained cyclooctene or an activated alkene; Thiol–ene consists of a thiol (sulfhydryl group) reacting with an alkene (olefin) to form a carbon–sulfur bond, typically in the presence of a photoinitiator or a radical initiator; Photo-click reactions are a collection of click reactions performed by the light application (a chromophore and multiple wavelengths are required).

Beyond CuAAC, the concept of click chemistry established by Sharpless encompasses a miscellaneous assembling of reactions like thiol–ene [6] and inverse electron-demand Diels–Alder (IEDDA) [7], promoted by radical and strained alkene/alkyne, respectively, with the former extensively used in polymer chemistry and material science, and the latter in bioconjugation and bioorthogonal chemistry. Subsequently, directed by the inconvenience of metals’ presence in biomaterials [8], copper-free methodologies, such as strain-promoted azide–alkyne cycloaddition (SPAAC) and strain-promoted alkyne–nitrone cycloaddition (SPANC), assisted by annular tension appeared [9]. All these chemical reactions promote conventional covalent bond formation but with the advantages of efficiency, selectivity, orthogonality, and swiftness that distinguish this chemical paradigm [10] (Figure 1).

The click reactions have allowed the development of new materials with applications in strategic areas such as the environmental [11,12,13], energy [14,15], and medicine (medicinal chemistry, drug development and delivery, tissue engineering, etc.), where increasingly more sophisticated systems are required in terms of specificity, multifunctionality, bioavailability, delayed clearance, multi-targeting, specific cytotoxicity, and tracking [16,17,18,19,20]. In this field, CuAAC has been the most used [21,22,23,24], followed by the metal-free versions, SPAAC and thiol–ene reactions [25,26,27].

Click chemistry has become an effective synthetic strategy for drug conjugation, bioconjugation, and surface functionalization, where the steric hindrance negatively affects the efficiency, kinetics, selectivity, and yields [28].

This review is organized into three sections to emphasize the click chemistry supremacy to overcome the significant challenge of functionalizing sterically hindered molecular systems such as polymeric molecules (Section 2), cyclodextrins (Section 3), and fullerenes (Section 4), with inherently voluminous groups like proteins, peptides, polysaccharides, DNA, or dendrons to obtain novel materials with potential therapeutic applications.

The successful modification of these molecular systems can be highly illustrative of the versatility of click chemistry compared to other conventional chemical reactions.

## 2. Click Reactions for Functionalization of Polymeric Systems

The post-polymerization modification of polymeric systems to tailor their properties is a significant challenge, as many polymerization reactions are sensitive to the functional groups originally present. Furthermore, side-chain modification of polymers with large numbers of ligands requires highly efficient coupling reactions that overcome steric hindrance and account for the limited solubility of many polymers in post-functionalization reactions. Therefore, click chemistry has attracted tremendous attention in polymer post-synthesis protocols [29,30,31,32], even in inhomogeneous reaction systems, including topochemical reactions in the solid state [33].

Since early 2007, W.H. Binder and R. Sachsenhofer reviewed the application of click reactions for post-derivatization of various polymers, oligomers, biopolymers, and gels initially obtained by atom-transfer radical polymerization (ATRP), ring-opening metathesis polymerization (ROMP), and quasi-living cationic polymerization or polycondensation reactions [34].

Nowadays, click chemistry has positively impacted the pharmaceutical industry by the effective obtaining of polymer–biomolecule with varied structures like micelles or nanocapsules, among others [23,24].

Particularly, thiol–ene and thiol–yne click reactions have shown high versatility for the modification of polymer backbones through radical mechanisms using thermal or photochemical processes [23]. Despite higher steric hindrance in polymers, the click reactions proceeded fast, overcoming activation barriers [35]. As acknowledged, conventional covalent transformations are highly sensitive to steric environments where a slow molecular diffusion may be present. In the case of click reactions, the apparent effective handling of steric effects is the reason for the high stereoselectivity observed in the products. Sterically hindered conditions like those observed in polymeric systems, may promote stereoselective bond formation more efficiently [36].

### 2.1. Polymer–Biofunctionality Conjugates: Complexity in Scope and Size

**Figure 2:**
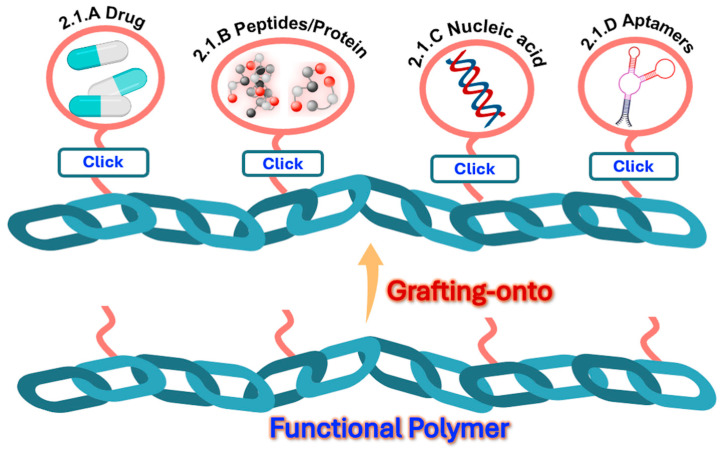
Conjugation scenarios involving “grafting-onto” polymers by click chemistry to obtain biomedically relevant systems.

In the medical area, the range of click reactions, including CuAAC, SPAAC, and thiol–ene metal-free click reactions, has allowed the obtaining of sophisticated molecular systems with improved delivery, targeting, and therapeutic efficacy. The “grafting-onto” approach has emerged as a versatile strategy for attaching bulky biofunctional groups to polymer backbones. Figure 2 illustrates the polymer conjugation scenarios discussed in this review, along with their corresponding section numbers.

Through the post-polymerization scheme, conjugates of polymer–drug, polymer–antibody, polymer–peptide/protein, polymer–nucleic acid, and polymer–aptamer (short DNA or RNA molecules that bind specific targets) have been reported. Table 1 summarizes the revised studies in which click reactions were used to modify polymeric platforms to obtain systems for different applications in nanomedicine.

#### 2.1.1. Polymer–Drug Conjugates

**Figure 3:**
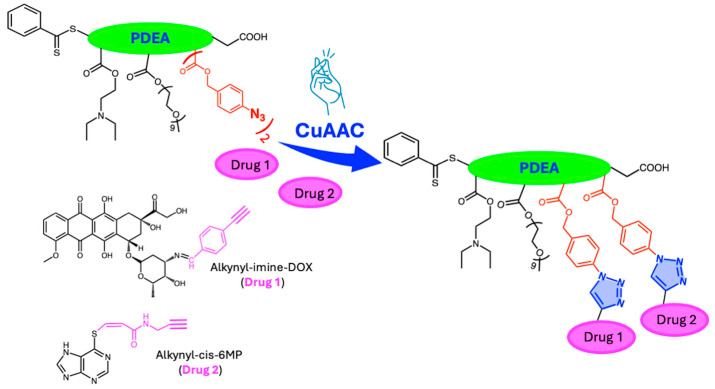
Illustration of the synthesis of PDPAO@imine-DOX/cis-6MP prodrugs.

Currently, multidrug resistance in diseases such as cancer is a significant challenge in the treatment of various malignancies. It has been shown that grafting polymers with various bioactive molecules promotes positive synergism to combat multidrug resistance and enables the incorporation of targeting mechanisms. Using CuAAC reaction, dual-drug-conjugated prodrugs, denoted as PDPAO@imine-DOX/cis-6MP (2.1 A, Figure 2), was synthesized by grafting doxorubicin (DOX), a well-known chemotherapeutic agent with a broad spectrum of anticancer activities, and 6MP, another well-known anticancer drug utilized as an antimetabolic agent in the treatment of human leukemia, onto poly(2-(diethylamino)ethyl methacrylate) (PDEA) selected as base component of the nanocarriers (Figure 3) [37].

After 48 h, the dual-drug conjugate was obtained with a precise Drug1/Drug2 ratio based on the controlled stoichiometric feeding ratio of the drugs. The dual-drug platform could self-assemble into polymeric micelles, facilitating internalization. The drugs’ synergistic effects were demonstrated in vitro against both HeLa and HL-60 cells, highlighting the importance of having efficient protocols, such as those of click chemistry, to assemble increasingly complex therapeutic systems.

Nguyen et al. [38] synthesized conjugates between hydrophilic polymers based on methoxy-PEG2000 and the anti-tumor drug, paclitaxel (PTX), by the CuAAC reaction, with a yield of 55 to 60% (2.1 A, Figure 2). In addition to the inherent impediments imposed by the polymer, the steric hindrance of the group neighboring the ester bond, potentially cleavable during the drug release, was modulated to appraise the effect on the release rate and the corresponding efficacy. The conjugate with moderate steric hindrance promoted sustained and increased PTX release, resulting in enhanced anti-tumor efficacy.

In addition to facilitating the incorporation of drug molecules, the orthogonality and performance of click reactions have allowed the one-pot integration of other important functions, like targeting and imaging, onto polymeric chains, despite the sterical challenge involved.

**Figure 4:**
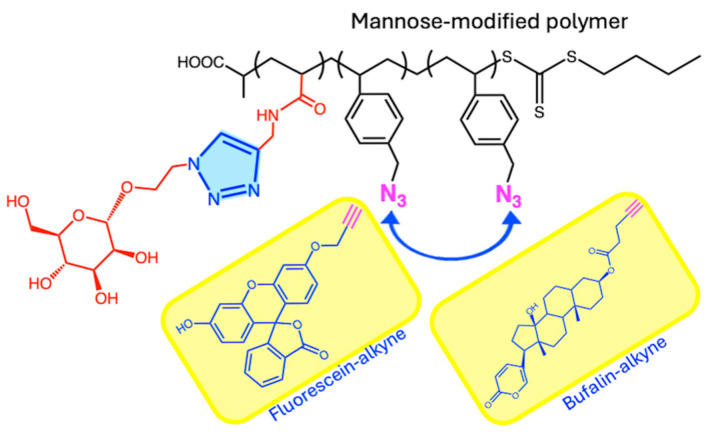
Illustration of the synthesis of a multifunctional polymeric conjugate containing bufalin and fluorescein through one-pot coupling on polymeric chains via the CuAAC reaction.

A multi-function polymeric conjugate, containing targeting, imaging, and therapeutic moieties, was prepared by the combination of reversible addition–fragmentation transfer (RAFT) polymerization and click chemistry [39]. An acrylamide monomer containing mannose units for targeting was synthesized by CuAAC reaction, and copolymerized by RAFT in the presence of 2,2’-azobis [2-(2-imidazolin-2-yl)propane]dihydrochloride and 1-(azidomethyl)-4-vinylbenzene, thus incorporating azide pendant units. Meanwhile, the bioactive bufalin (natural steroid with anticancer properties), as well as fluorescein, were functionalized with alkyne groups for posterior one-pot coupling on the polymeric chains by CuAAC reactions (2.1 A, Figure 2). The multi-function conjugate was obtained in a 50% yield (Figure 4).

The cellular uptake behavior of this polymeric conjugate was studied by flow cytometry, where its effective entry into cells was observed, thus improving the drug (bufalin) internalization. In vitro cell viability assays performed in HepG2 cell cultures (related to liver cancer) evidenced a lower non-specific toxicity and tumor uptake of the multi-function conjugate, which showed controlled release for potential clinical applications.

An amphiphilic stimuli-responsive polymeric prodrug, based on polyphosphoester grafted with RGD-PEG-N_3_ (RGD, tumor marker peptide for hepatocellular carcinoma cells) and camptothecin, CPT-N_3_ (CPT, anti-cancer alkaloid), was synthesized in one-pot via the CuAAC reaction (2.1 A and 2.1 B, Figure 2) [40]. Both active molecules, previously azide-functionalized, were sequentially clicked onto a hydrophilic polymer for enhanced targeting and enrichment in tumor tissue. The double substitution was carried out with a 55% yield. The polymeric prodrug exhibited a CPT loading capacity (wt%) of up to 15%. As disulfide bonds were included in the CPT moieties, their easy cleavage in the tumor environment of liver cancer cells allowed the controlled release of the CPT drug, exhibiting lower non-specific toxicity.

**Figure 5:**
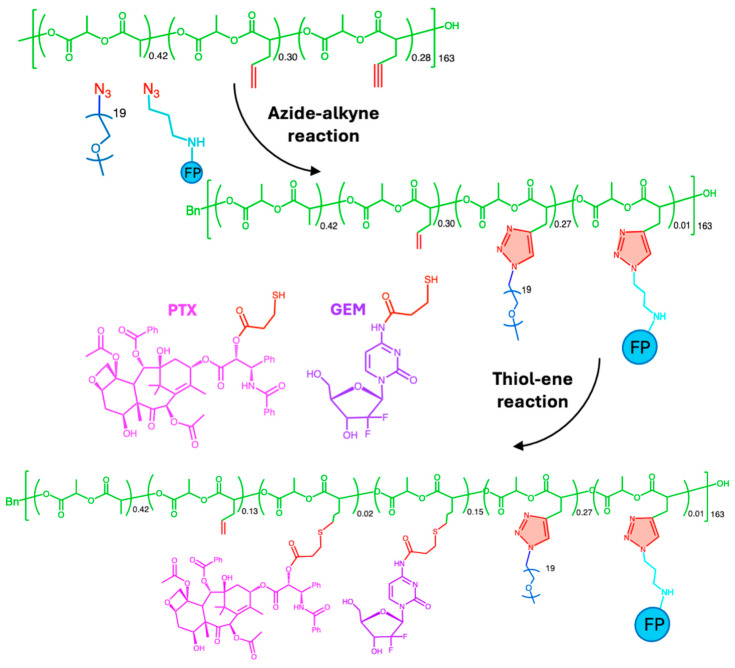
Azide–alkyne and thiol–ene reactions to conjugate PEG chains, FP, PTX, and GEM.

The incorporation of linkers during conjugation has been a suitable strategy to outperform steric hindrance. Recent advances in linker design have led to the creation of click chemistry-based linkers, significantly improving the production of highly stable and site-specific polymer–drug conjugates [53]. Polymer–drug conjugates based on polyethylene glycol (PEG) were reported with different linkers to ensure a regulated drug release [54]. The combination of linkers on the same polymeric backbone allows the conjugation of two or more different drugs to achieve a co-delivery scenario. Su et al. reported a polylactide-graft-poly(ethylene glycol) (PLA-g-PEG) polymer combinedly conjugated with two anticancer drugs (2.1 A, Figure 2), hydrophobic PTX and hydrophilic gemcitabine (GEM) by hydrolysable ester-thioether (–OCOCH_2_CH_2_S–) and amide-thioether (–NHCOCH_2_CH_2_S–) clickable linkers, respectively (Figure 5) [41]. Fluorophore (FP) was also included for imaging purposes. Azide–alkyne and UV-induced thiol–ene click reactions were employed to conjugate PEG chains, FP, PTX, and GEM, taking advantage of their chemoselectivity and orthogonality. The resultant conjugate was obtained in a 95.9% yield. According to the ^1^H-NMR results, conjugation efficiencies of 4.1 wt% for PTX and 8.1 wt% for GEM were achieved.

#### 2.1.2. Polymer and Peptides/Proteins

The synthesis of polymer–protein or polymer–peptide conjugates is of great importance in fields related to biotechnology. Combining the properties of polymers and biological molecules like peptides and proteins can enhance the stability, solubility, and bioactivity of the resulting systems. Furthermore, the conjugation of polymers with proteins (peptides) may improve the pharmacokinetics of drugs by extending their circulation time in the bloodstream, reducing immunogenicity, and enhancing target-specific delivery. Polymer conjugation also protects proteins and peptides from enzymatic degradation, ensuring their therapeutic efficacy over longer periods. The use of versatile synthetic strategies, such as click chemistry, enables precise control over the architecture of these conjugates, allowing for the development of more complex and multifunctional systems.

Using the common alternate copolymer poly(styrene-co-maleic anhydride) as a polymeric substrate, its functionalization with azidopropylamine allowed a subsequent bioconjugation with bovine serum albumin (BSA) (2.1 B, Figure 2), one of the most studied proteins in bioconjugation due to its accessibility and well-known structure, via strain-promoted azide–alkyne cycloaddition (SPAAC) reaction, a copper-free click chemistry reaction [45]. The grafting of BSA was up to 60%, despite the steric hindrance inherent to the size and shape of this protein.

Protein A is a bacterial protein, originally found in the cell wall of *Staphylococcus aureus*, that has great importance in the field of biotechnology and immunology due to its ability to selectively bind antibodies for their purification and further diagnostic applications.

Azide-functionalized Protein A ligands were covalently clicked by CuAAC reaction onto a polymeric matrix of regenerated cellulose that was previously modified by grafting copolymers of the alkyne monomer propargyl methacrylate (PgMA) and the spacer monomer poly(ethylene glycol) methyl ether methacrylate (PEGMEMA300) at varying PgMA-*co*-PEGMEMA300 compositions to obtain membranes (2.1 B, Figure 2) [42]. The highest Static Binding Capacity (SBC) of the clicked membranes, understood as the maximum specific binding to a chromatographic medium under non-flow conditions, occurred at 50% (*w/w*) PgMA-co-50% PEGMEMA300. The incorporation of a high number of specific interaction sites through the successful integration of protein A for antibody purification in chromatographic processes, overcoming steric hindrance, highlights the effectiveness of click reactions.

The conjugation of antibodies (specialized proteins produced by the immune system) with stimulus-responsive polymers has attracted attention as a strategy to improve the sensitivity for detecting biomarkers at low concentrations. Steric hindrance is a critical factor to consider in polymer–antibody conjugation because it can significantly affect the functionality (affinity), binding efficiency, and overall performance of the conjugates.

**Figure 6:**
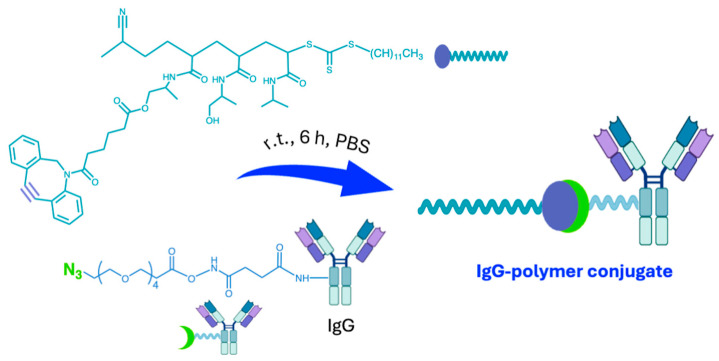
Preparation of sterically hindered antibody-temperature-responsive polymer conjugate via SPAAC click reaction.

Hironaka et al. developed a successful complex polymeric system for biomarker enrichment, designed to promote antibody–antigen recognition and synthesized it in situ by click chemistry. The azide functional group was previously incorporated into Immunoglobulin G (IgG), which is composed of four peptide chains, facilitating high specificity in the conjugation between the polymeric moiety and the antibody–antigen complexes (2.1 B, Figure 2) [46] (Figure 6). First, azido-IgG was mixed with a urine specimen to enable antigen–antibody interaction, followed by conjugation with an alkyne-group-modified temperature-responsive polymer using click chemistry, despite the congestion imposed by the presence of macromolecules.

Hydrogels are sterically demanded three-dimensional hydrophilic polymer networks that can be engineered as smart materials for numerous applications including drug delivery systems, wound care, tissue engineering, and regenerative medicine [55]. The use of common click reactions, including CuAAC, SPAAC, thiol–ene, and IEDDA reactions, has been conducive to the preparation of biomedical hydrogels and their subsequent functionalization [51,56,57].

**Figure 7:**
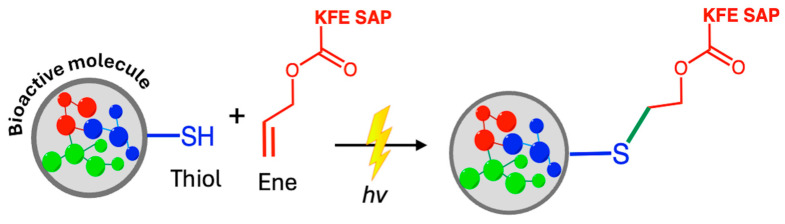
Thiol–ene click chemistry reaction to covalently bind a thiolated bioactive molecule and KFE self-assembling peptide (SAP).

Some types of hydrogels formed by self-assembling peptides (SAP) are used as models for cancer research and as tissue engineering scaffolds. Further functionalization with particular amino acid sequences allows the incorporation of specific biological signals. Covalent functionalization through click reactions, such as thiol–ene initiated by UV light, allows control of the exact positions of moieties’ incorporation before gel formation. Peptide sequence KFE (acetyl-FKFEFKFE-CONH_2_), used to form biomedical hydrogels, was covalently functionalized by click chemistry (2.1 B, Figure 2), introducing a fluorescent label, an integrin binding site, and a matrix metalloproteinases (MMPs)-sensitive biosensor to track the degradation of extracellular matrix (ECM) proteins, crucial in tissue remodeling, wound healing, and cancer metastasis (Figure 7) [51].

The efficient clicking of various functional molecules did not disrupt either the fibrous microarchitecture of the SAP or the mechanical properties of the resulting hydrogel. These smart SAP hydrogels supported cell encapsulation with high viability and provided spatiotemporal control of functionalization with biomolecules and biosensors.

Moreover, a thiol–ene click chemistry protocol between maleimide and thiol was applied as the final cross-linking step to generate 3D-bioprintable and biocompatible hydrogels based on α-elastin, an important extracellular matrix protein (2.1 B, Figure 2), and hyaluronic acid [52]. Contrary to reported protocols for elastin covalent cross-linking using classical chemistry, such as the carbodiimide method, which impacts cell viability due to by-products derived from unreacted linkers, the authors exploited the biocompatibility of this click reaction to form tunable hydrogels, suitable for 3D bioprinting, under mild reaction conditions (3 h at 37 °C).

#### 2.1.3. Polymer–Nucleic Acid Conjugates

Several click-chemistry approaches have been applied for post-polymerization modifications with nucleic acids, such as DNA and RNA, to afford advanced biomaterials with applications in gene delivery, diagnostics, and tissue engineering [58,59]. The covalent conjugation of nucleic acids to polymer backbones generates covalent polyplexes that increase system stability in biological environments, improve release control, allow for the inclusion of targeting ligands, and reduce toxicity.

**Figure 8:**
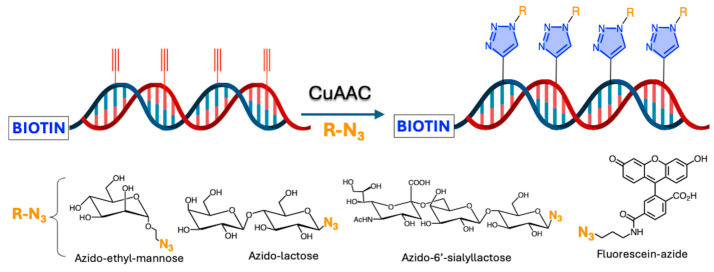
Azido-modified molecules conjugated to a DNA template.

A DNA template based on 5-(octa-1,7-diynyl)uracil 20-deoxy-20-fluoroarabinonucleic acid (FANA) triphosphate was functionalized with contiguous alkynyl groups to enable efficient click conjugation with voluminous azide-functionalized molecules (2.1 C, Figure 2), thereby becoming a promising platform for serving as a programmable and evolvable synthetic genetic polymer capable of post-polymerization functionalization (Figure 8) [43].

The DNA–polymer exhibited excellent resistance to nuclease degradation, without compromising the polymerase recognition. The efficiency and mildness of the click protocol provide a valuable platform for presenting a variety of chemical functionalities including carbohydrates, fluorophores, and hydrophobic or charged moieties.

Using synthetic polymers like poly(ethoxyethyl glycidyl ether) (PEEGE) and poly(ε-caprolactone) (PCL), two linear amphiphilic nucleic acid–polymer conjugates (NAPCs) were synthesized. These included poly(ethoxyethyl glycidyl ether)-block-poly(propylene oxide)-block-oligonucleotide conjugate and oligonucleotide-block-poly(ε-caprolactone)-block-oligonucleotide conjugate (2.1 C, Figure 2), via click coupling reactions under mild conditions (40 °C for 48 h). The azide group was incorporated as N_3_-oligonucleotides, and the alkynyl group was comprised of polymer-C≡CH [44]. The resulting NAPCs exhibited amphiphilic behavior and formed stable aggregates in aqueous solution, as a result of efficient functionalization, displaying non-toxicity and biocompatibility, which enhanced cellular uptake without the need for additional transfection agents.

To guarantee a high density of nucleic acids on the surface of the molecular systems to exploit the recognition properties of Deoxyribonucleic Acid (DNA) moieties, a rational design of the polymer–oligonucleotide conjugates’ shapes is needed. Spherical 3D nucleic acid–polymer conjugates of comb-like and coil-comb chain architectures were obtained by grafting multiple alkyne-functionalized oligonucleotide strands (5−8 oligonucleotide strands per polymer chain) onto azide-modified homo-, block, and random copolymers of chloromethylstyrene via initiator-free click coupling. This reaction can be used to create semi-interpenetrating polymer networks, for example, via Bergman cyclization (2.1 C, Figure 2) [47]. The resulting conjugates are amphiphilic and form stable nanosized supramolecular structures in aqueous solution. The steric hindrance became evident as the resistance to nuclease degradation increased with higher grafting density. The 3D nucleic acid nanostructures exhibited valuable properties for drug delivery, molecular diagnostics, gene regulation, and various nucleic acid-based therapeutic approaches.

#### 2.1.4. Polymer–Aptamer Conjugates

Aptamers, also known as “chemical antibodies”, are short, single-stranded oligonucleotides (DNA or RNA) that can specifically bind to a wide range of targets, including proteins, small molecules, and even whole cells, with high affinity and selectivity. Polymer–aptamer conjugation is highly relevant for advancing drug delivery systems, biosensors, and therapeutics, as it addresses major limitations such as rapid clearance from the body by reducing renal elimination and preventing enzymatic degradation.

Azide PEG-like polymer brush (Mw ≈ 40 kDa) was conjugated with 31-nucleotide RNA aptamers by SPAAC under mild conditions (24 h at room temperature) (2.1 D, Figure 2) [48]. The synthetic protocol enabled site-specific and stoichiometric (1:1) conjugation without reacting with any other chemical groups on the aptamers. This conjugate does not induce a humoral immune response against the polymer itself in mice and preserves the RNA therapeutics.

**Figure 9:**
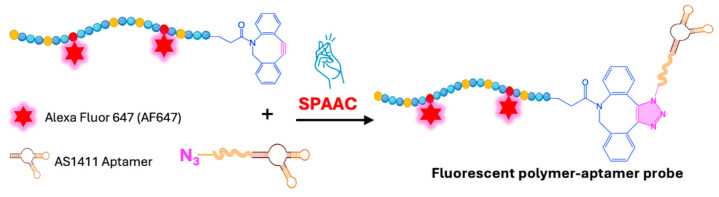
Polymer−aptamer conjugation by SPAAC between DBCO polymer chain and azido-functionalized aptamer.

Conjugation via SPAAC between dibenzylcyclooctyne (DBCO)-ended fluorescent polymer chains and 3′-azido-functionalized nucleic acids was carried out at 37 °C to obtain polymer–aptamer probes (2.1 D, Figure 2) able to provide selective and super-resolved detection of cell surface nucleolin, a multifunctional protein involved inessential biological processes (Figure 9) [49]. DBCO is widely used for bioconjugation and chemical labeling due to its commercial availability and its irreversible and selective profile of reactivity with azides to form stable triazole linkages through click reactions like SPAAC [60].

The incorporation of DBCO promoted conjugation yields of up to 60%, using a polymer/aptamer molar ratio of 10 and a reaction time of 72 h. The extension of reaction time had the greatest impact on conjugation yield. Compared with other tested synthetic approaches, SPAAC conjugation between DBCO-terminated fluorescent polymer chains and 3′-azido-functionalized nucleic acids proved to be the most efficient.

High-drug-loading Aptamer−PolyproDrug Conjugates (ApPDCs), designed for targeted combination cancer therapy, were synthesized by conjugating cancer cell-targeting aptamers with copolymerized polyprodrugs from the monomers SN-38M (ethyl-10-hydroxycamptothecin (SN-38)) and PTXM (paclitaxel, PTX), each with distinct pharmacological mechanisms, onto a (poly(ethylene glycol) (PEG)-like brush polymer, named P(OEGMA), as the polymeric substrate (2.1 D, Figure 2) [50]. The drug molar ratio SN-38/PTX in the final polyprodrugs was azide-P(OEGMA_0.75_-*co*-SN-38M_0.125_-*co*-PTXM_0.125_)_320_. The 1/1 ratio foreseen in the design ensures the synergistic anticancer effect. Subsequently, DBCO-modified DNA and azide-terminated polyprodrug were dissolved in deionized water and reacted under the SPAAC reaction conditions at 37 °C for 24 h. The used aptamer Sgc8c specifically targets the human protein tyrosine kinase PTK-7, which is primarily expressed on the human colon cancer cell line (HCT116) and the human acute lymphocytic leukemia cell line (CCRF-CEM). All the bulky modules (drugs, brush polymer, and aptamers) were successfully clicked under mild conditions to obtain molecular systems with improved anticancer bioactivity.

## 3. Functionalization of Cyclodextrins

Other molecular platforms such as cyclodextrins have been successfully functionalized with bulky substituents [9], using click reactions for the final coupling. The next section focuses on the reactivity features of CDs, mainly centered on their hydroxyl groups. Their functionalization with biologically active molecules, applying the precision of click chemistry in sterically challenging environments, allows for the development of advanced biomaterials and drug delivery systems.

Cyclodextrins (CDs) are cyclic oligosaccharides composed of glucose units linked by α-1,4 glycosidic bonds. Depending on whether they are comprised of six, seven, or eight glucose units, CDs are named α-, β-, and γ-cyclodextrin, respectively. CDs have a unique structure consisting of a hydrophilic outer surface and a relatively hydrophobic central cavity able to form inclusion complexes with guest molecules of appropriate size and shape through non-covalent interactions, such as hydrogen bonding and hydrophobic interactions. The solubility and stability of guest molecules with therapeutic activity are enhanced after complexation with CDs. Therefore, these molecular platforms—especially βCD—hold significant importance in pharmaceutical sciences, where major efforts are directed toward improving the solubility, stability, and bioavailability of bioactive molecules [61].

Beyond the applicability of native βCD, its derivatives, such as hydroxypropyl (HPβCD) and sulfobutylether (SBEβCD), demonstrate enhanced solubility, thermal stability, biocompatibility, and stimuli-responsive behavior, thereby broadening the applications of these molecules [62,63].

**Figure 10:**
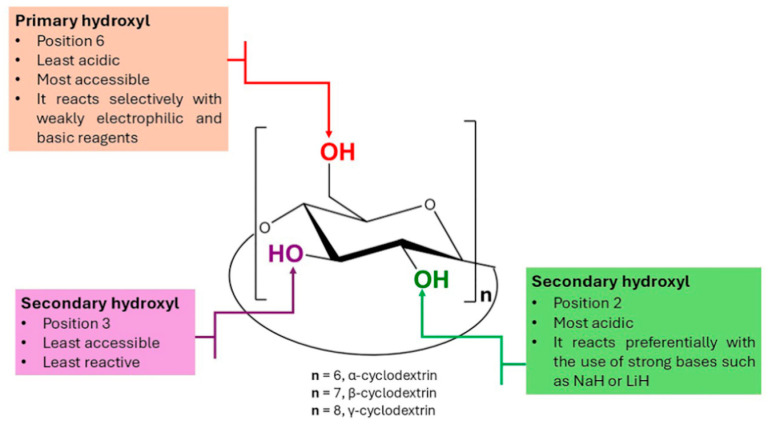
Reactivity of cyclodextrins.

The precise functionalization of CDs by the chemical modification of their different hydroxyl groups presents a significant challenge due to the steric hindrance imposed by their rigid, cone-like structure, which creates spatial constraints around them [64,65] (Figure 10).

From all hydroxyl groups of CDs, primary ones are generally more reactive than secondary ones, and the difference in reactivity is most likely due to steric control [66]. Moreover, secondary hydroxyl groups on positions 2 and 3 are involved in intra- and intermolecular hydrogen bonding, thus further reducing their reactivity compared to the primary hydroxyl groups.

**Figure 11:**
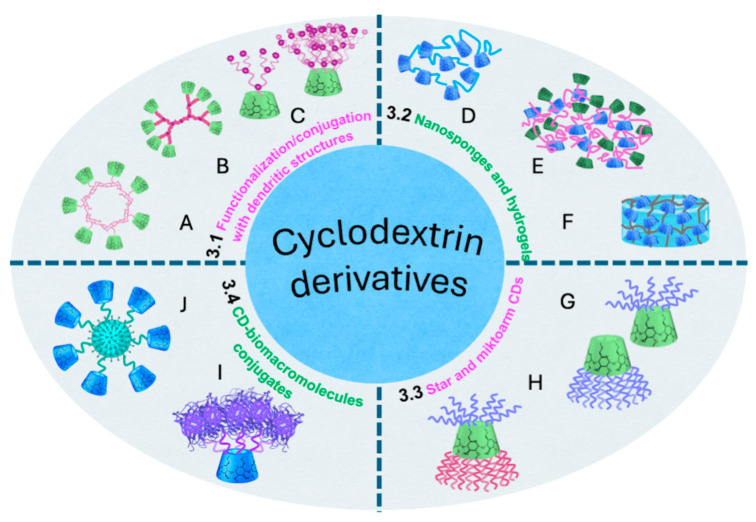
Schematic representations of different CD derivatives synthesized via click chemistry. 3.1 Dendrimer–CD conjugates (scenarios A, B, and C), 3.2 Nanosponges and hydrogels (scenarios D, E, and F), 3.3 Star and miktoarm CDs (scenarios G and H), 3.4 CD–biomacromolecule conjugates (scenarios I and J). All scenarios are described above.

Combining the particular reactivity of CDs with the broad range of possibilities offered by click chemistry has led to diverse CD-based systems with high versatility in biomedical and pharmaceutical applications [67]. Figure 11 provides an overview of the CD-based structures discussed in this review. The revised arrays involving CD include dendritic structures (Section 3.1), where CD is found as the core and as a peripheral terminal group (A), where it is only the peripheral terminal group (B), and where a CD core is functionalized with dendrons of different type (C). Additionally, three CD-based nanosponge and hydrogel systems were considered (Section 3.2): (D) cross-linked, (E) hyper-cross-linked, and (F) CD hydrogels. In Section 3.3, star and miktoarm CD architectures are mentioned, where equal-length chains were attached to the central CD core on both primary and secondary faces (G), or where a combination of equal and unequal chains were attached to the CD core (H). Finally, CD–biomacromolecule conjugates (Section 3.4), where CD may be the core (I) or the peripheral group (J). These systems, obtained through coupling bulky molecular motifs and CDs using click chemistry protocols, emphasize their efficiency in overcoming steric hindrance.

Table 2 highlights the CuAAC reaction as the predominant click chemistry approach for synthesizing CD derivatives tailored to drug delivery systems. However, copper-free click reactions, such as SPAAC and thiol–ene, are gaining increasing significance in the development of these materials. The table summarizes the studies discussed in this section, organized according to the categories presented in Figure 11.

### 3.1. CD Functionalization/Conjugation with Dendritic Structures

Combining dendritic structures with CDs (3.1 systems, Figure 11) has been explored to create innovative systems that promote synergy between both materials to improve, for instance, drug encapsulation efficiency, enabling controlled release [89]. Nevertheless, their coupling represents a scenario of considerable steric hindrance due to the bulkiness of the dendrons and the three-dimensional arrangement of hydroxyl groups within the rigid CD framework [90,91]. To address this difficulty, molecular linkers are often used to reduce spatial constraints [92]. The use of linkers applying click chemistry protocols has been successfully merged in the combination of dendritic architectures with CDs. These studies are detailed below.

**Figure 12:**
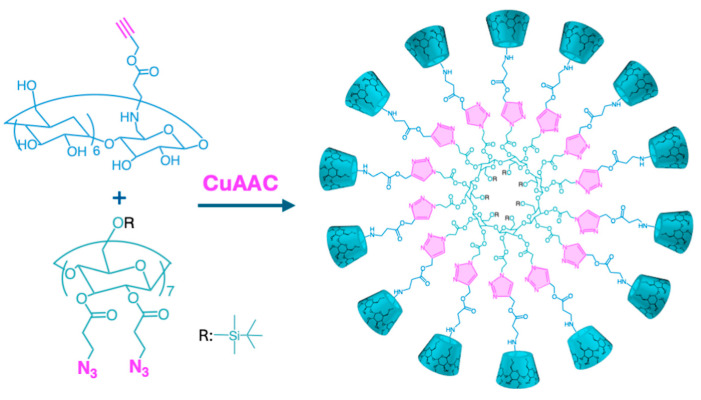
βCD dendrimer with 14 βCD units attached to the βCD core synthesized via CuAAC coupling.

Toomari et al. [68] synthesized a βCD dendrimer (3.1 A, Figure 11) by attaching seven βCD units to the primary face of a βCD core through CuAAC click reaction, using β-alanine propargyl ester hydrochloride as a molecular linker. Utilizing CuBr/2,2′-bi-pyridine as a catalytic system in Dimethylformamide (DMF), the product was obtained in a 53% yield. The resulting βCD dendrimer exhibited an encapsulation efficiency of 79.8% for the anticancer drug methotrexate (MTX), significantly improving over the 52% achieved with native βCD. This result was attributed to the combined entrapment within the βCD cavity and the dendritic branches. The in vitro toxicity of the synthesized βCD dendrimer and its inclusion complex with MTX was assessed on T47D epithelial cells using the MTT test. Results showed that the synthesized βCD dendrimer was not cytotoxic to the cell line, while the IC_50_ values for free MTX and the MTX-βCD dendrimer complex on T47D cells were 7.4 and 4.9 µM, respectively. Additionally, in vitro drug release studies in buffer solution demonstrated that the βCD dendrimer promoted a more controlled release of the loaded MTX over 72 h, compared to 23 h for MTX loaded in native βCD. Furthermore, when 14 βCD residues were linked to the βCD core on the secondary face (3.1 A, Figure 11), a 45% yield was achieved using the same synthetic method (Figure 12). This modification enhanced the encapsulation efficiency of MTX up to 84.7% while reducing the IC_50_ to 3.2 μM, showcasing the effectiveness of the synthesized dendrimer for controlled drug delivery systems for cancer treatment [69]. These studies demonstrated the successful synthesis of well-defined CD dendrimers with high encapsulation efficiency achieved through click reactions while retaining biocompatibility via βCD. Other drugs, including naproxen, naltrexone [93], and DOX [94], were successfully encapsulated in CD dendrimers synthesized through click reactions.

Chen et al. [70] reported the synthesis of multivalent Mucin 1 (MUC1) glycopeptide dendrimers (3.1 C, Figure 11) based on βCD using the CuAAC click reaction with CuBr/thioanisole as a catalyst system in DMF, achieving a 59% yield. Seven MUC1 glycopeptide units were attached to the primary face of the per-6-azido-βCD core using a bifunctional PEG-based linker with an alkynyl group at the N-terminus of the peptides. This strategy was crucial for overcoming steric hindrance caused by the highly glycosylated MUC1 units. The resulting heptavalent glycopeptide dendrimer exhibited enhanced recognition and association with antibodies and epitopes, owing to the multivalent effects. The binding strength of MUC1 glycopeptide dendrimers to a multivalent epitope was nearly five times greater than to a monovalent epitope, highlighting a pronounced multivalent effect with promising applications in antibody detection, serum analysis, and disease diagnosis.

Sorroza-Martinez et al. [71] successfully functionalized the entire periphery of PAMAM dendrimer G1 with βCD units using the CuAAC click reaction (3.1 B, Figure 11). Despite the steric challenges of attaching eight βCD units, they achieved an 80% yield within 48 h. This efficient synthesis method emphasizes the versatility of CuAAC for constructing complex dendritic architectures. The resulting βCD-based PAMAM dendrimer exhibited a significant increase in water solubility (620.5 mg/mL), much higher than native βCD. This enhanced solubility, combined with its non-cytotoxic effects in cancer cell lines (MCF-7, MDA-MB-231, and HeLa), underscores the potential of this dendritic system for drug delivery applications.

**Figure 13:**
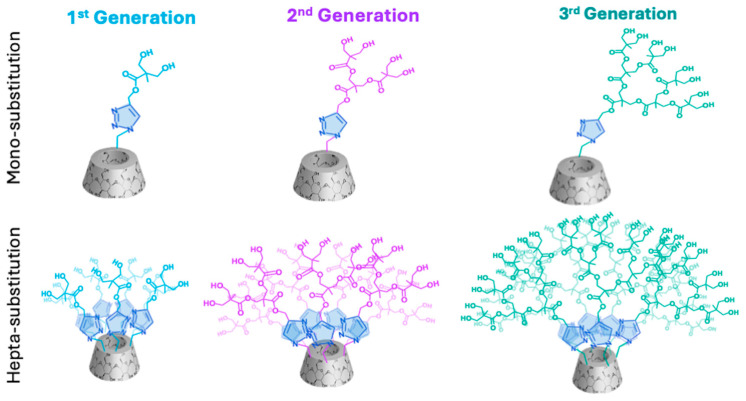
Mono- and hepta-substituted poly(ester) dendritic βCD derivatives from first to third generation obtained by CuAAC reaction.

López-Méndez et al. [72] synthesized poly(ester) dendritic βCD derivatives (3.1 C, Figure 11), modifying two key structural variables: the dendritic generation (from first to third) and the degree of substitution on the βCD primary face (mono- or hepta-substitution), using the CuAAC reaction (Figure 13). Despite substantial steric hindrance due to varying generation sizes and degrees of substitution, well-defined dendritic βCD derivatives, monodisperse and pure, were successfully synthesized, achieving yields ranging from 70 to 89%. These dendritic derivatives demonstrated enhanced solubility and drug-loading capacities, compared to native βCD, particularly for albendazole (ABZ) as the guest molecule, showing a clear correlation between the dendritic generation and increased encapsulation efficiency. The obtained molecular platforms appear to be promising materials for improving ABZ solubility, thus positively impacting its therapeutic desirability [95].

### 3.2. CD-Based Nanosponges and Hydrogels

Nanosponges and hydrogels based on CDs (3.2 systems, Figure 11) have garnered significant attention in biomedical and pharmaceutical research due to their versatile applications, particularly in drug delivery [96].

The synthesis of nanosponges typically involves the use of cross-linking agents such as chlorohydrins, acid anhydrides, diisocyanates, acyl chlorides, and carbodiimides [97]. However, these methodologies often require an excess of cross-linking agents and result in low yields, likely due to the close arrangement of CD units within the nanosponge’s reticulated structure, which can hinder efficient cross-linking [98,99]. In this context, click chemistry emerges as a promising alternative to enable a more effective synthesis of these systems, facilitating a controlled and efficient cross-linking process.

Meo et al. reported the synthesis of βCD–calixarene nanosponge copolymers via the CuAAC reaction between two different heptakis-6-azido-CDs and two different propargyloxy-calix[4]arenes. In this study, both molecules functioned as hosts and cross-linkers simultaneously (3.2 D, Figure 11). Different proportions of molecules were chosen to obtain materials with an excess of azide and alkyne functional groups, achieving yields of up to 96% [73]. These nanosponges were subsequently subjected to chemical transformation with amine or carboxylic ionizable groups. The adsorption properties of the nanosponge as a function of pH exhibited a complex behavior, with the triazole groups showing an increased proton-binding capacity due to the formation of intranetwork hydrogen bonds. These compounds represent an effective approach to preparing nanosponge-based nanocarriers with the capacity to encapsulate two or more drugs with diverse properties [100].

Degirmenci et al. reported the synthesis of responsive nanogels containing βCD for targeted drug delivery. These nanogels were prepared from βCD-functionalized dextran and bis-adamantane-crosslinker containing disulfide moieties, which are responsive to glutathione (GSH), a molecule over-expressed in many cancer cells [74]. The βCD-functionalized dextran polymer (3.2 F, Figure 11) was synthesized via CuAAC reaction between propargyl-functionalized dextran and an azide-containing βCD derivative, catalyzed by CuBr and pentamethyldiethylenetriamine (PMDETA) in DMF for 25 h at 50 °C, achieving yields of up to 69%. Nanogel formation involved self-assembly in aqueous media of the βCD–dextran polymer with the disulfide-containing bis-adamantane-crosslinker. The nanogels were then loaded with DOX, an anticancer drug, and adamantane-functionalized cyclic RGD peptides (arginine-glycine-aspartic acid), allowing for targeting binding to integrin receptors overexpressed on cancer cells. DOX release was responsive to pH and redox stimuli, with maximal release occurring under acidic, GSH-rich conditions. The empty nanogels showed no cytotoxicity, while DOX-loaded nanogels displayed cytotoxic effects against MDA-MB-231 breast cancer cells. This efficient synthetic approach shows promise for targeting various cancers through stimuli-responsive drug delivery systems.

Although CuAAC remains widely used for nanosponges and hydrogel synthesis, recent focus has shifted towards metal-free click transformations. This shift arises from concerns that residual metal impurities could compromise the biological function of the resulting materials. Additionally, metal-free click reactions have gained significant interest due to their high reactivity and selectivity under mild reaction conditions [26].

**Figure 14:**
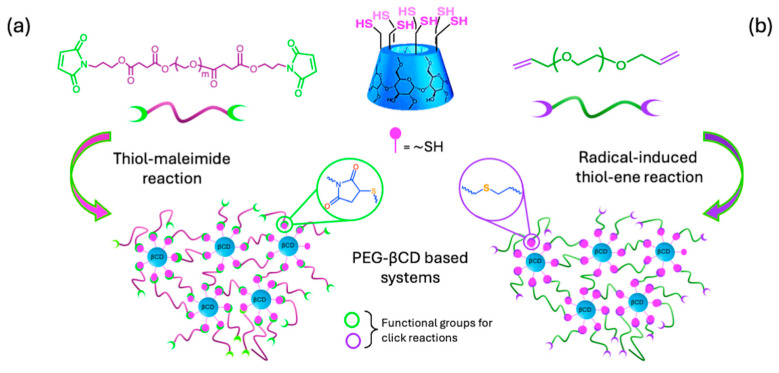
Preparation of PEG–βCD systems via (**a**) thiol–maleimide conjugation and (**b**) radical thiol–ene conjugation.

In this regard, Arslan et al. [84] synthesized a PEG-based system incorporating βCD using the thiol–maleimide click reaction (3.2 E, Figure 11). Despite the steric hindrance from bulky polymer chains and crowding around the cross-linking sites, which could limit access to certain βCD regions, they successfully achieved gel conversions up to 92% under mild reaction conditions. The synthesis involved maleimide-containing telechelic PEGs that reacted with thiol per-functionalized βCD (Figure 14a) in the presence of a catalytic amount of triethylamine and DMF as the reaction medium. By adjusting the functional group (either thiol or maleimide) stoichiometry, they produced hydrogels capable of post-gelation functionalization, as demonstrated by the attachment of fluorescent dyes *N*-(5-fluorosceinyl)maleimide and thiol-containing fluorescein, allowing for variability to tune the extent of their functionalization as desired. This method provides controlled incorporation of βCD into hydrogels, expanding the possibilities for host-guest interactions, with potential applications in areas such as tissue engineering.

Subsequently, the same research group detailed the synthesis of a system using bifunctional linear PEGs with allyl groups and heptavalent thiol-functionalized βCD (3.2 E, Figure 11) via the radical-induced thiol–ene click approach (Figure 14b) [85]. The reaction occurred under UV irradiation (365 nm) with the photoinitiator 2,2-dimethoxy-2-phenylacetophenone (DMPA) in DMF with PEGs of varying molecular weights (2 kDa to 8 kDa). Higher conversion rates were observed with shorter PEG chains, as longer chains introduced steric hindrance that reduced gel conversion. Despite this, the method consistently yielded soft, transparent materials with up to 91% conversion. Morphological analysis revealed that pore sizes increased with the length of the PEG chains, while all materials demonstrated favorable swelling behavior, driven by the hydrophilic nature of the PEG chains. Additionally, these systems successfully encapsulated the hydrophobic drug puerarin, used for glaucoma treatment, and achieved controlled drug release through the ICs with βCD. These findings suggest that the synthesized hydrogels hold significant promise for the development of advanced controlled drug delivery systems.

A chitosan–βCD hydrogel (3.2 F, Figure 11) was synthesized through a Diels–Alder reaction between furfural functionalized chitosan (CF) and *N*-maleoyl alanine functionalized HPβCD (HPβCD-AMI) in aqueous solution, without the need for any catalyst or initiator by the research group of Zhang et al. [88]. Characterization of the hydrogel revealed a stable, flexible network, as confirmed by rheological analysis, which showed a high storage modulus (*G*′), ranging from 1 to 1200 Pa. Depending on the application, this range is ideal for ensuring the hydrogel remains stable without being rigid or brittle, which is crucial for its ability to encapsulate and release drugs over an extended period, minimizing premature release or collapse in biological environments [101]. The increase in *G*’ corresponds to the rising degree of furfural substitution on chitosan, from 2.6% to 28.3%, suggesting that the hydrogel’s mechanical strength can be effectively tuned by adjusting the reaction stoichiometry. Drug release studies using methyl orange demonstrated a controlled release profile, with only 48.85% of the drug released after 24 h in a PBS buffer, highlighting the hydrogel’s potential for controlled drug delivery.

On the other hand, Zhang et al. [86] synthesized βCD-based hydrogels in an aqueous solution via thiol–maleimide click reaction (3.2 F, Figure 11) using thiolated-βCD (βCD-(SH)_7_) and *N*-maleoyl alanine-functionalized HPβCD (AMI-HPβCD) with a degree of substitution (DS) of hydroxypropyl in HPβCD of 8 and DS of *N*-maleoyl alanine in AMI-HPβCD of 2.3. The bulky structure of *N*-maleoyl alanine-functionalized HP-βCD posed a challenge due to steric hindrance, potentially leading to incomplete binding to thiolated-βCD. Therefore, the use of thiol–maleimide click chemistry at elevated temperatures (up to 80 °C) was relevant for ensuring complete and stable bonding during hydrogel formation, resulting in a high molecular weight (up to Mw of 4.11 × 10^5^) and star-like branched structure, with yields reaching up to 52%. Hydrogels with high M_w_ typically feature denser polymeric networks, which enhance their mechanical stability and resist rapid degradation under physiological conditions. This characteristic is essential for applications where the hydrogel must maintain its shape and functionality for extended periods, such as in implants or controlled drug-release systems [102]. Additionally, in these hydrogels, βCD not only serves as a structural backbone but also as a host for hydrophobic drug molecules. The release behavior of curcumin, a hydrophobic antioxidant with anticancer activity, was evaluated, revealing that approximately 80% of the curcumin (over 350 min) was released from βCD hydrogel, with a slower release rate compared to native βCD. The interconnected hyperbranched network of these hydrogels demonstrated excellent controlled-release properties, making them promising candidates for drug delivery and pharmaceutical applications.

### 3.3. Star and Miktoarm CDs

Star polymers consist of several equal or unequal (miktoarm star) linear chains linked together at the central core, where the number of arms is determined by the functional capacity of the core molecule. The compact, globular architecture and high arm density of multiarm star systems make them attractive for a wide range of biomedical applications, encompassing drug and gene delivery, tissue engineering, diagnostic tools, antibacterial biomaterials, and bioimaging [103].

CDs represent ideal central scaffolds due to their multiple hydroxyl groups; however, the dense arrangement of these functional groups makes difficult the full core substitution, therefore affecting the synthetic reproducibility of these systems, which impacts their desired properties [104]

One of the most widely used methodologies for synthesizing these systems is ATRP [105], though it presents several challenges, such as low yields, partial substitutions, and the use of catalysts that are toxic [106,107,108].

Click chemistry has emerged as a straightforward and robust method for achieving multivalent functionalization of CDs, enabling the creation of star-type or miktoarm CD structures. This approach permits not only the derivatization with polymeric arms but also large biological molecules, significantly enhancing the potential of these structures for various biological and biomedical applications. In the evolution of the methodologies for the synthesis of these systems, click chemistry may be applied alone or in combination with ATRP.

Wang et al. developed a nanocarrier based on a βCD-core star copolymer with poly(ethylmethacrylate) (PEMA), PEG, and poly(2-(diethylamino)ethyl methacrylate) (PDEA) blocks (3.3 H, Figure 11) [75]. The synthesis combined ATRP and CuAAC reactions. Initially, βCD with 21 initiation sites corresponding to all hydroxyl groups served as the core for ATRP, using CuCl/CuCl_2_/hexamethyl-triethylene-tetramine (HMTETA) as the catalytic system to introduce PEMA blocks with azide groups, obtaining CD-star-PEMA-N_3_. Subsequently, PEG and PDEA blocks previously functionalized with alkyne groups were incorporated by CuAAC reaction, achieving quantitative yields. The resulting copolymer was self-assembled into micelles in aqueous solution, where the PEMA moieties acted as a reservoir for hydrophobic guest molecules, PEG provided hydrophilicity, and the PDEA block introduced pH sensitivity. These micelles effectively encapsulated hydrophobic compounds such as celecoxib, and ketoprofen. Thus, amphiphilic macromolecules like those described above show considerable potential for drug delivery systems with pH-responsive release mechanisms.

Gou et al. synthesized a miktoarm-like system by combining an ATRP reaction to modify the secondary face of βCD and the CuAAC reaction to derivatize the primary face. In this approach, initially was protected the most reactive primary face with *tert*-butyldimethylsilyl groups, followed by a controlled ring-opening polymerization of ε-caprolactone on the secondary face. Then, ibuprofen was covalently attached to the terminal hydroxyl groups of the poly(ε-caprolactone) chains by the Steglich esterification reaction. To introduce azide groups needed to CuAAC reaction, the primary face was deprotected, followed by acylation and direct azidation with NaN_3_, instead of the more commonly used iodination–azidation strategy. Finally, alkyne-terminated PEG was coupled by a click reaction with a 90% yield to produce the desired amphiphilic miktoarm star copolymer (3.3 H, Figure 11) [76].

Xu et al. reported the synthesis of well-defined 7-arm and 21-arm poly(*N*-isopropylacrylamide) (PNIPAM) star polymers, utilizing βCD as a core [109]. To obtain these systems, first, heptakis(6-deoxy-6-azido)-βCD and heptakis[2,3,6-tri-*O*-(2-azidopropionyl)]-βCD were synthesized, and then they were coupled with linear alkyne-PNIPAM via CuAAC click reaction (3.3 A, Figure 11). This strategy allowed the obtaining of low-polydisperse products, predominantly exhibiting a complete substitution of the βCD core. The temperature-responsive behavior of these systems, by PNIPAM units, may be valuable for various biomedical applications like bioseparation and tissue engineering [110].

A star βCD polymer (3.3 H, Figure 11) was reported by Li et al. as a Magnetic Resonance Imaging (MRI) contrast agent with positive imaging contrast enhancement and suitable longitudinal relaxivity (T_1_), ideal for soft or fatty tissues. It was made starting from a βCD-based core consisting of 7 azide groups in the primary face and 14 α-bromopropionate moieties in the secondary face. ATRP was used to substitute the secondary face with polycationic poly(*N*,*N*-dimethylaminoethyl methacrylate) (PDMA) arms, and CuAAC click reaction was used to substitute the primary face with a 1,4,7,10-tetraazacyclododecane-1,4,7,10-tetrakis acetic acid Gd complex. This combined approach allowed 78% substitution of the secondary face and total substitution of the primary face in a quantitative manner using click chemistry [77].

Rojas-Aguirre et al. reported the synthesis of three distinct PEGylated βCD star-shaped polymers (3.3 G, Figure 11) using PEG 5000 and PEG 2000 as gold-standard physicochemical stabilizers for drug carriers, and PEG 550 as a low molecular weight polymer model. The synthesis involved coupling alkynyl-PEG with hepta-substituted azide βCD on the primary face via the CuAAC click reaction. Complete hepta-substitution was achieved with yields of approximately 90%. The interaction of these systems with bio-interfaces, including Vero cells, HeLa cells, and peripheral monocytes, was systematically investigated for potential application in drug delivery systems. The findings of this work showed that the PEG 550-βCD derivatives significantly reduced the viability of HeLa cells and human monocytes, highlighting that combining molecular platforms originally innocuous does not necessarily yield materials with the same biological behavior as their precursors [78].

A significant contribution to the synthesis of star CD derivatives using click chemistry is presented by Yi et al. [87]. They utilized thiol–ene photoclick chemistry to develop multifunctional precursors derived from fully functionalized βCD cores (3.3 H, Figure 11), leading to the formation of multiarm precursors. The synthesis process involved dissolving perallylated-βCD and 2,2-dimethoxy-2-phenylacetophenone (DMPA) in thiol 2-mercaptoethyl-2-bromo-2-methylpropanoate (HS-EBiB) and exposing the mixture to UV irradiation at 365 nm for 30 min at room temperature without stirring. This methodology achieved an impressive 95% yield without requiring an inert atmosphere. The modular capabilities of thiol–ene photoclick chemistry enabled the synthesis of multifunctional cores based on αCD and γCD, producing 18-arm and 24-arm structures, respectively, from allyl-αCD and allyl-γCD, with yields exceeding 90%. The study shows that thiol–ene photoclick chemistry efficiently produces CD-based multifunctional initiators. These CD scaffolds initiate branched polymers that self-assemble into micelles, enhancing the solubility, bioavailability, and targeted delivery of hydrophobic drugs, making them valuable for biomedical applications.

Research has demonstrated that conjugating CDs with bulky biological molecules, such as peptides and proteins, offers significant advantages for therapeutic applications. These CD conjugates exhibit enhanced stability and bioactivity, making them highly versatile in drug delivery systems, targeted therapies, biomedical applications, and biosensing technologies [111].

**Figure 15:**
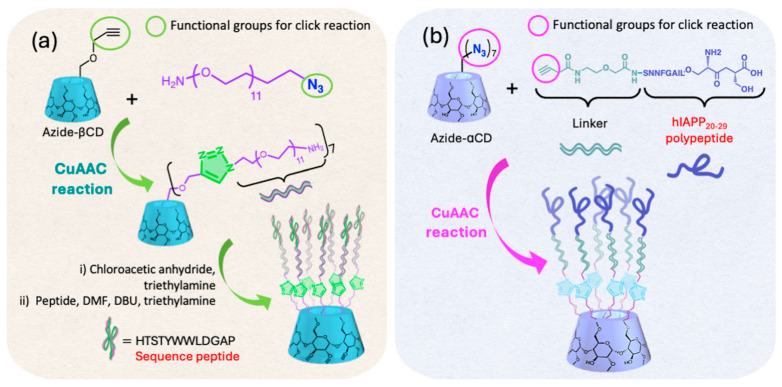
Representation of (**a**) heptavalent anthrax toxin inhibitor and (**b**) six peptide fragments attached to αCD.

Concerning the improvement of the peptide’s bioactivity, Joshi et al. [79] designed and synthesized a heptavalent inhibitor targeting anthrax toxin based on a methylated-βCD core (3.3 G, Figure 11). Using CuAAC reaction, seven copies of the anthrax-neutralizing peptide sequence (HTSTYWWLDGAP) were conjugated onto the primary face of a methylated-βCD, with PEG11 linkers to improve flexibility (Figure 15a). This conjugation process was conducted over 24 h at 80 °C, achieving an 86% yield. The heptavalent toxin inhibitor demonstrated remarkable efficacy, neutralizing anthrax lethal toxin in vitro and in vivo, with over a 100,000-fold enhancement in activity compared with the unconjugated peptide. Given the inherent biocompatibility of both βCD and PEG, this potent heptavalent inhibitor is a promising complement to antibiotic therapies in the treatment of anthrax.

Christoffersen et al. [80] reported the conjugation of alkyne–amyloid polypeptide fragment (hIAPP_20-29_) with azide-functionalized αCD (3.3 G, Figure 11) through CuAAC click reaction to investigate the fibrillation kinetics related to type 2 diabetes. The coupling was conducted in Dimethylsulfoxide (DMSO), using CuI at 60 °C for 3 h (Figure 15b), achieving an 84% yield. Their experiments revealed that the peptidic conjugates fibrillated more rapidly than the free peptide fragments. Moreover, these conjugates formed thinner and longer fibrils, while the free fragments produced thicker and twisted ones, suggesting that multimeric scaffolding could influence amyloid fibril formation pathways. Understanding how different peptide configurations affect fibrillation, particularly in the context of hIAPP fibrils that accumulate in pancreatic islets and damage the β-cell function in type 2 diabetes, could lead to strategies to control or prevent these deposits and may contribute to the development of therapies for amyloid-related diseases.

### 3.4. CD–Biomacromolecule Conjugates

Click chemistry for conjugating biomacromolecules with CDs has been a successful strategy to obtain valuable materials for biomedical applications like drug delivery, diagnosis, and the development of cell-based therapies.

**Figure 16:**
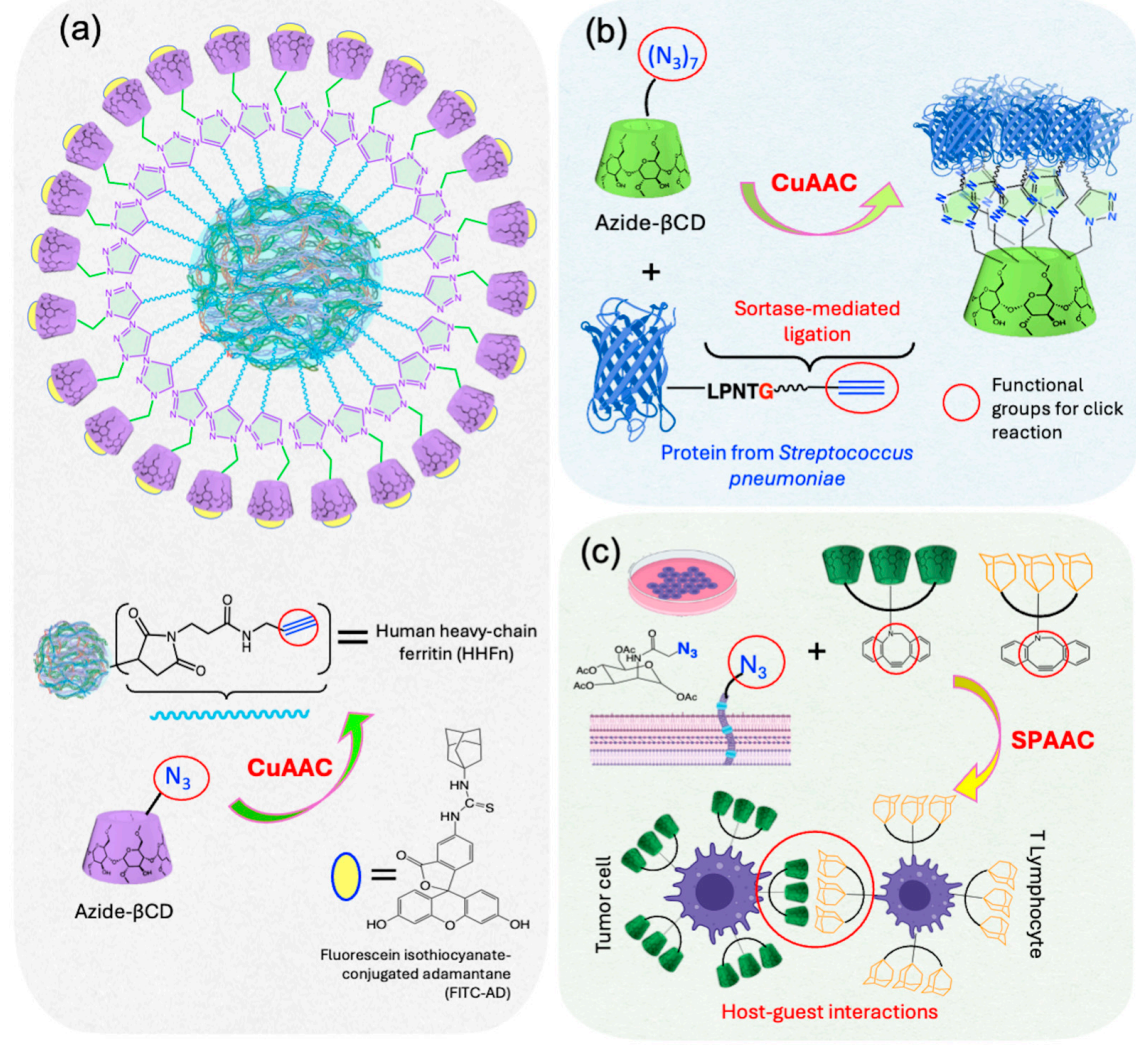
(**a**) Attachment of βCD motifs on the surface of HHFn through CuAAC reaction followed by the inclusion complex formation with FITC-AD; (**b**) conjugation of alkyne-labeled protein by sortase ligation and azide-βCD by CuAAc reaction; (**c**) cell surface modification via SPAAC ligation to cell–cell adhesion through host-guest interactions.

Kwon et al. [81] reported a method to synthesize nanoscale cargo delivery vehicles based on the modification of human heavy chain ferritin (HHFn) (Figure 16a), a protein containing cysteine subunits that were functionalized with propargyl groups through a thiol–maleimide click reaction. Subsequently, a CuAAC click reaction was used to conjugate 24 azide-functionalized βCD units to the propargyl-modified HHFn (3.4 J, Figure 11), achieving a yield of over 90% despite the significant steric hindrance associated with attaching multiple βCD units to a single HHFn. This synthetic strategy resulted in HHFn-βCD protein cages able to form ICs with fluorescein isothiocyanate-conjugated adamantane, allowing for controlled and gradual release around 3 h. The highly efficient conjugation method demonstrates the potential of these nanoplatforms for drug delivery, especially in transporting insoluble drugs, while also improving their use in diagnostics and imaging with fluorescent probes and contrast agents.

Singh et al. [82] reported the efficient synthesis of βCD conjugates with pneumococcal surface protein A (PspA) and RlrA-regulated gene B (RrgB) proteins (from *Streptococcus pneumoniae*) using CuAAC click reaction, achieving yields of around 70% in 1 h at room temperature (Figure 16b). The process involved alkyne labeling of proteins through the enzymatic sortase-mediated ligation, followed by their reaction with azide-βCD derivatives (3.4 I, Figure 11). Remarkably, despite the inherent steric hindrance that typically limits the attachment of multivalent protein units, this approach enabled the conjugation of up to seven protein units (PspA or RrgB) to a single βCD molecule. This innovative approach not only overcomes steric challenges but also highlights the potential of heptavalent CD–protein conjugates for future applications in medicine such as targeted therapies and co-delivery systems.

Plumet et al. [83] synthesized artificial recognition markers consisting of three units of βCD, each attached to a DBCO group for coupling to azido-labeled cell surfaces via a SPAAC click reaction (3.4 J, Figure 11). This approach enabled the synthesis of markers that promoted unnatural cell–cell adhesion. A549 and Jurkat T cells were first treated with tetraacetylated *N*-azidoacetyl-D-mannosamine (Ac4ManNAz) for 3 days, allowing azide groups to be incorporated into their membranes without affecting cell viability for at least 48 h post-functionalization. Subsequently, the cells were incubated with the βCD units for 30 min using the SPAAC reaction, effectively bypassing the spatial challenges imposed by the large and rigid βCD structures. The trimeric design provided multiple recognition sites for host-guest interactions, significantly improving cell–cell adhesion, which was confirmed by scanning electron microscopy, revealing Jurkat T cells adhering to A549 tumor cells (Figure 16c). These synthetic surface markers offer versatile tools for linking different cell types, paving the way for advanced studies of intercellular interactions and contributing to the development of cell-based therapies and deeper insights into adhesion mechanisms.

## 4. Functionalization of Fullerenes

**Figure 17:**
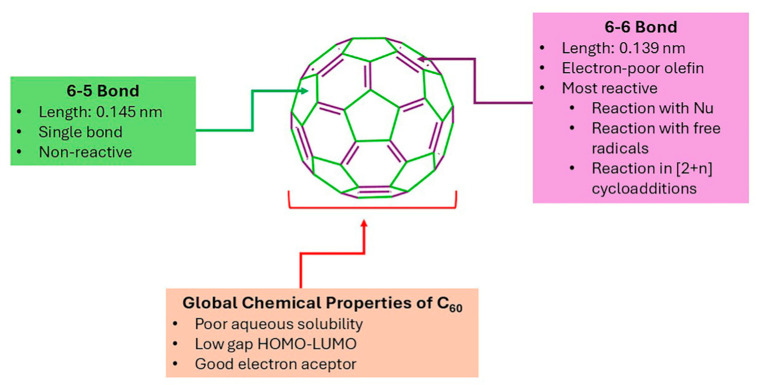
Structural and reactivity characteristics of C_60_.

In 1985, Smalley and colleagues synthesized a spherical carbon allotrope called fullerene, named for its resemblance to geodesic domes designed by the American architect Fuller [112]. C_60_ is the most extensively studied fullerene with such structural characteristics that exhibits 60 non-reactive single bonds, alongside 30 reactive localized double bonds (Figure 17) [113]. The reactivity of C_60_ is attributed to the strain present in the non-planar double bonds, which is relieved during the transition of carbon hybridization from sp_2_ to sp_3_. From a chemical perspective, C_60_ resembles an electron-deficient alkene [114], prone to react with nucleophiles (Nu) [115], free radicals [116], and in [2 + *n*] cycloadditions (*n* = 1, 2, 3, 4) with, for example, dienes and 1,3-dipolar species [117]. Among these transformations, the most studied is the [2 + 1] cycloaddition of 2-halo-1,3-dicarbonyl compounds promoted by strong bases known as the Bingel-Hirsch (B-H) reaction. This reaction gives cyclopropane derivatives known as methanofullerenes [118].

Fullerene C_60_ and its derivatives have been intensively investigated in medicine against various diseases, as anticancer agents [119,120], antimicrobial [121,122,123], antivirals against HIV [124], and SARS-CoV-2 [125], and for the treatment of neurodegenerative diseases [126]. Despite the large number of applications of C_60_, one of its main drawbacks limiting its general use in medicine is its hydrophobic nature [127], which affects its biocompatibility [128]. Derivatization of C_60_ is a widely used strategy to enhance their aqueous solubility and biocompatibility, as well as to improve their overall properties. However, the process can be particularly challenging or even impossible when dealing with sterically hindered molecules, especially in the synthesis of hexa-adducts. In this regard, click chemistry has emerged as a valuable approach, facilitating the coupling of bulky structures with ease. This method employs straightforward and adaptable protocols that do not necessitate harsh conditions, resulting in favorable yields. Click chemistry has proven to be crucial in the development of novel materials and biologically active molecules derived from C_60_ moieties [129].

Before delving into notable works on the click derivatization of C_60_, it is important to emphasize that the molecule’s inherent reactivity can pose challenges. This reactivity may compete with certain components that are commonly employed in click reactions. For example, azides can react with C_60_ without the presence of copper catalysts due to the electron-deficient nature and the double bond strain in fullerene, which promote the [2 + 3] cycloaddition, generating a 1,2,3-triazoline ring [130].

**Figure 18:**
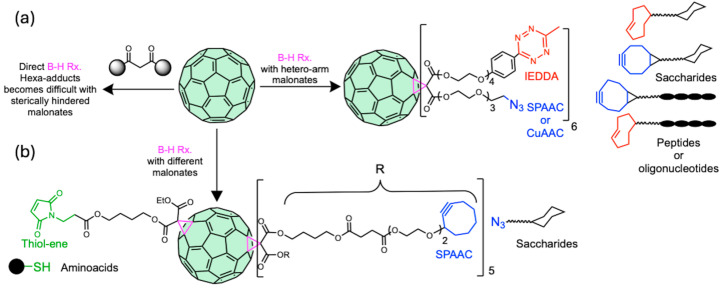
Combining B-H reaction with click chemistry for the C_60_ conjugation: (**a**) orthogonal use of IEDDA and CuAAC, (**b**) orthogonal use of SPAAC and thiol-ene.

A common strategy to successfully derivatize C_60_, avoiding undesired products, has been the combination of B-H and click synthetic methodologies. In this sense, the use of hetero-arm or different malonic ester substrates in the B-H reaction allows post-functionalization through different click reactions. For example, it has been possible to derivatize C_60_ orthogonally by means of SPAAC and IEDDA reactions with terminal azides and tetrazines for the controlled conjugation with biomolecules such as peptides, oligonucleotides, and/or monosaccharides [131] (Figure 18a). In a complementary study, C_60_ with terminal cyclooctyne and maleimide functions was conjugated with saccharides and amino acids through the SPAAC and thiol–ene reactions respectively [132] (Figure 18b).

**Figure 19:**
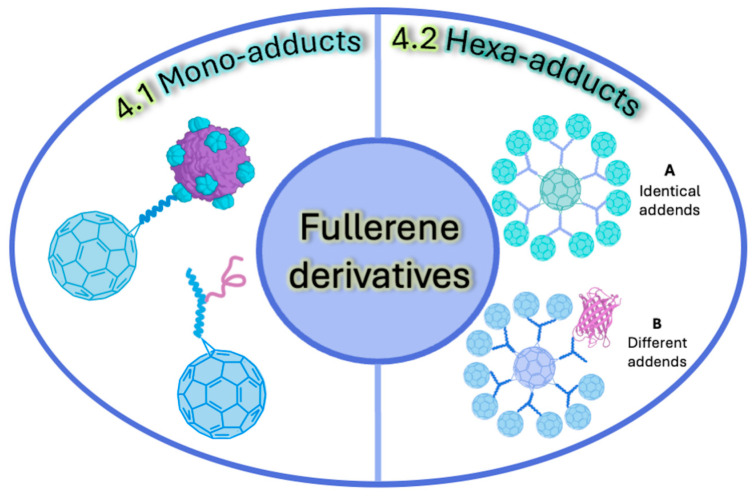
Classification of C_60_ derivatives as a function of the number of substituents: 4.1 mono-adducts, with a single addend attached to one of their carbon atoms, and 4.2 hexa-adducts, functionalized with six addends, either identical (A) or different (B).

In the subsequent section, we will describe the utilization of click chemistry for the coupling between C_60_ and bulky bioactive motifs focusing on improving the performance of these materials in biomedical applications. Two general scenarios were identified in the preparation of bioactive C_60_ derivatives; in Section 4.1, we will describe the use of mono-adducts, and in Section 4.1.1 and Section 4.1.2, hexa-adducts will be covered, which are divided into derivatization with identical addends (A) and with different addends (B) (Figure 19).

As depicted in Table 3, the CuAAC reaction has become the primary click chemistry approach for developing C_60_-based macromolecular systems aimed at treating bacterial and viral infections. However, the SPAAC and thiol–ene methods are gaining prominence, broadening the range of functional groups employed in C_60_ derivatization. Table 3 summarizes the studies covered in this section and categorizes them based on Figure 19.

### 4.1. Mono-Adducts of Fullerene

The primary drawback of C_60_ derivatization lies in the difficulty of controlling the number of additions, which often results in low yields of mono-adducts (4.1 systems, Figure 19). Typically, the B-H reaction can produce between 1 and 6 additions of malonic acid derivatives. In this context, the formation of multi-adducts leads to stereoisomeric mixtures, complicating their separation [146]. While the yields of mono-adducts typically do not exceed 45%, many of these derivatives have been synthesized. Nonetheless, we have identified only one example of click chemistry derivatization of mono-adducts involving sterically hindered biological systems.

**Figure 20:**
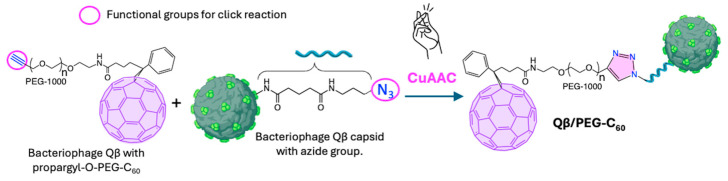
Mono-functionalization of propargyl-O-PEG-C_60_ with a bacteriophage Qβ through CuAAC click reaction.

In this regard, Steinmetz et al. [133] reported the conjugation of a C_60_ derivative to viral nanoparticles (VNPs), specifically the capsid of bacteriophage Qβ, using a CuAAC click reaction (4.1, Figure 19). The propargyl-O-PEG-C_60_ derivative was attached to azide-functionalized bacteriophage Qβ at room temperature for 2 h, yielding Qβ/PEG-C_60_ conjugate with enhanced aqueous solubility. In this conjugate, the bacteriophage Qβ unit served as a hydrophilic motif, making C_60_ water-soluble. At the same time, the PEG linker introduced through the CuAAC reaction mitigated the steric hindrance posed by the bulky bacteriophage Qβ, demonstrating its effectiveness in overcoming spatial challenges when attaching large biomacromolecules (Figure 20). To evaluate its biomedical potential, Alexa Fluor^®^-568 fluorophore was attached to Qβ/PEG-C_60_. Cellular uptake assays confirmed that the conjugation of the Qβ capsid to the C_60_ unit did not hinder internalization. Therefore, this strategy may be suitable for developing scaffolds or delivery vehicles in cells, with promising applications in tumor therapies and novel therapeutic devices.

#### 4.1.1. Identical Addends (A)

**Figure 21:**
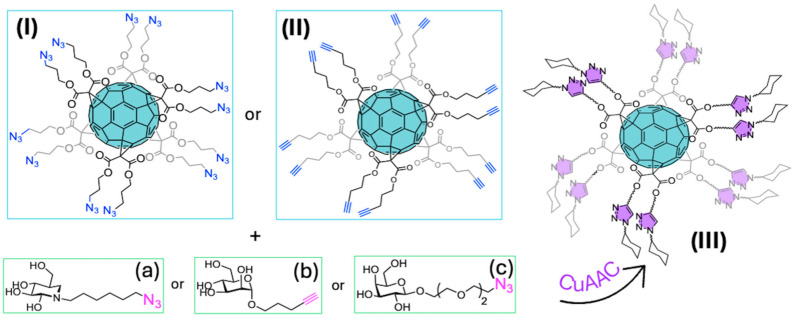
Development of hexakis-adducts of fullerene (**III**) with (**I**) 12 azide functions or (**II**) 12 alkyne functions for further derivatization through CuAAC click reaction using (**a**) azide-iminosugar 1-deoxynojirimycin, (**b**) alkyne-D-mannose and, (**c**) azide-D-galactose.

Nierengarten et al. developed the synthesis of novel C_60_ hexa-adducts with twelve azide (I) [147] or alkyne (II) [148] terminal groups (Figure 21). Then, they conducted their conjugation with carbohydrate derivatives using the CuAAC reaction, demonstrating the efficiency of this methodology in obtaining robust and highly functionalized systems like glycofullerenes (III) (Figure 21) [149,150].

These types of conjugates increase the local carbohydrate concentration, promoting multivalent effects by enhancing the number of binding sites to lectins, glycosidases, and glycosyltransferases, thereby improving their therapeutic potential [151].

In their initial works, Nierengarten et al. synthesized hexakis adducts of the iminosugar 1-deoxynojirimycin (Figure 21a) as glucosidase inhibitors by the CuAAC reaction between a Trimethylsilyl (TMS) protected dodeca-alkynyl fullerene and the corresponding azide-iminosugar. The in situ deprotection of TMS was performed with Tetrabutylammonium fluoride (TBAF), and the click reaction with the catalytic system of Cu_2_SO_4_⋅5H_2_O and sodium ascorbate at room temperature in a mixture of Dichloromethane (DCM)/H_2_O/DMSO, giving a very good yield of around 80% [152]. This multivalent system (IIIa) showed better activity compared to the monovalent system in the inhibition of β-galactosidase of the bovine liver, α-galactosidade of green coffee, β-glucosidase of almonds, α-mannosidade of Jack Bean, among others. Then, they expanded their investigation into lectin interactions to inhibit bacterial infections. The mannose glycofullerene (IIIb) was used as an inhibitor of bacterial adhesion through the Fimbriae D-mannose specific adhesin (FimH) lectin of *Escherichia Coli*. The multivalency of this globular structure showed enhanced affinities (in the nanomolar range) compared with the monovalent system [134]. In a posterior study, these authors developed the synthesis of galactose and glucose dodecavalent glycofullerenes through CuAAC reaction, using the same reaction conditions described above, obtaining a remarkable 86% yield. These derivatives were tested with PA-IL, a lectin present in *Pseudomonas aeruginosa* [135]. After the evaluation of the saccharide-protein affinity was observed that among glycofullerenes, the galactose derivative (IIIc) exhibited an enhanced activity compared with the glucose analogue, and both were more active than the monovalent saccharide derivatives.

**Figure 22:**
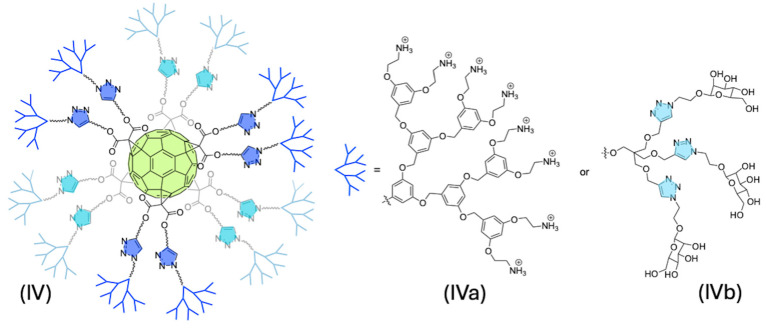
Preparation of dendrofullerenes (**IV**) through CuAAC reaction, obtention of polyether type dendrimer (**IVa**), and the glycodendrofullerene (**IVb**).

Different bulky structures like dendritic ones have been successfully attached to C_60_, giving gigantic systems with interesting biological properties [153]. In this context, Sigwalt et al. developed a hexa-adduct with 12 terminal TMS protected alkynes through the B-H reaction, to be further coupled to polyether dendrons with azide focal points. The in situ deprotection was performed as usual with TBAF, and then CuAAC reaction was carried out. As expected, the first-generation dendron gave a better yield of 83% compared with the second-generation dendron (IVa) (Figure 22) which gave an acceptable yield of 68%. The obtained dendritic structures with terminal ammonium groups were evaluated as gene delivery agents through their electrostatic interactions with plasmid DNA [154].

In another example, Rojo et al. developed water-soluble glycodendrofullerenes focused on tackling viral infections. The initial B-H synthesis to obtain C_60_ with 12 terminal alkynes was conducted, and it was subsequently coupled with an excess of an azide derivative of mannose by the CuAAC reaction, obtaining a spherical derivative with 12 sugar residues. To increase the number of mannose moieties, they synthesized two different dendrocarbohydrate azide addends based on pentaerythritol moiety. These dendrons present an azide function as a focal point and contain three sugar residues with either 1 or 3 ethylene glycol spacers. The coupling of these dendritic structures to the alkyne–fullerene was performed using CuBr-SMe_2_, Cu(0) in DMSO at room temperature to give a surprising 88% yield of glycofullerenes bearing 36 copies of mannoses on the periphery (IVb) (Figure 22). The binding abilities of these water-soluble compounds were tested with Concanavalin A, demonstrating the accessibility of these sugars to be recognized by a lectin. Then, they performed an infection in vitro assay with Ebola Virus Glycoprotein (EBOV-GP) pseudotyped viral particles as infectious agents. It was observed that a dependence of the inhibition effect on the number of mannoses may be established. The glycofullerene with 12 mannoses showed an IC_50_ of 2 μM, whereas the activity of the glycodendrofullerene with 36 mannoses was 68 μM (34-fold less active). It seems that the increase of the valency produced a decrease in the activity; nonetheless, the introduction of a longer spacer in glycodendrofullerene presents the recovery of the activity, with an IC_50_ of around 0.3 μM (a more than 200-fold increase in comparison with the 1-ethylene glycol spaced glycodendrofullerene) [138].

Ruiz-Santaquiteria et al. synthesized a C_60_ hexa-adduct peripherally decorated with twelve tryptophan (Trp) or tyrosine (Tyr) residues for potential use as a dual inhibitor against human immunodeficiency virus (HIV) and enterovirus 71 (EV71) (4.2 A, Figure 19) [136]. To reduce steric hindrance in the conjugation to the C_60_ scaffold (with twelve terminal alkyne groups), Trp and Tyr were modified with linkers of azide-PEG. The conjugation was performed via CuAAC in DMSO with CuBr at room temperature over 24 h, achieving yields of up to 84%. The resulting compounds showed significantly higher potency against HIV and similar activity against EV71, compared to similar prototypes with the same number of Trp or Tyr residues but attached to a smaller, more flexible pentaerythritol core. This highlights the importance of the globular 3D presentation of peripheral groups and the presence of spacers for effective virus interaction. The synthesized conjugates represent a new and versatile class of biocompatible carbon-based scaffolds, ensuring a unique globular distribution with promising potential as antiviral agents, particularly against HIV.

The synthesis of glycofullerenes containing 120 carbohydrate units on the periphery (4.2 A, Figure 19), targeting EBOV-GP inhibition, was reported by Muñoz et al. [137]. First, C_60_ hexa-adducts with twelve terminal alkyne or azide groups from simple malonates via B-H reaction were synthesized. Subsequently, twelve glycofullerenes containing ten mannopyranoside or galactopyranoside units (with the azide or alkyne group) were linked to C_60_ hexa-adducts, using CuAAC click reactions with CuSO_4_⋅5H_2_O and sodium ascorbate in Tetrahydrofuran (THF)/H_2_O, achieving yields of up to 79% despite the system’s complexity and large size. Biological studies showed that these glycofullerenes effectively inhibit EBOV-GP on Jurkat cells overexpressing Dendritic Cell-Specific Intercellular adhesion molecule-3-Grabbing Non-integrin (DC-SIGN), a critical pathogen-recognition lectin, with subnanomolar IC_50_ values. This highlights the effectiveness of multivalent carbohydrates in blocking cell-surface lectin receptors and emphasizes the need to manage steric effects in designing multivalent systems to ensure optimal ligand accessibility, pointing to a promising new antiviral strategy.

**Figure 23:**
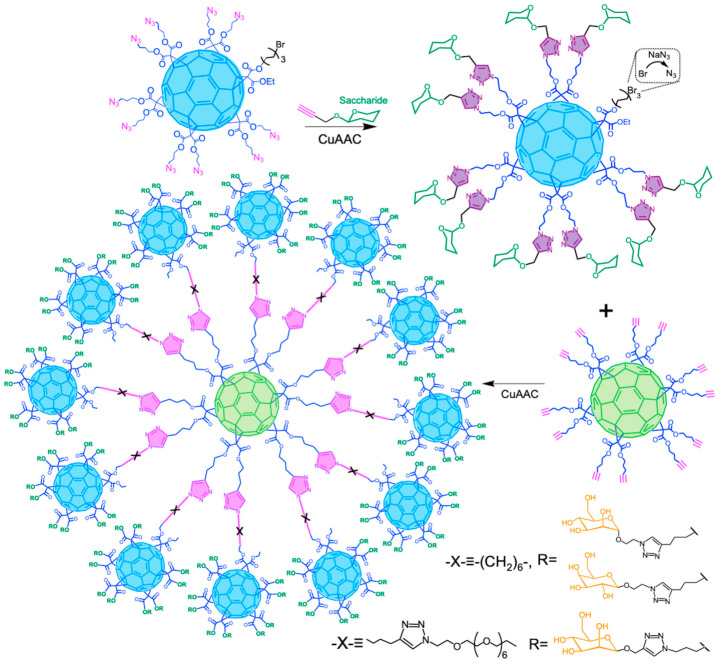
Gigant glycodendrofullerenes obtained by two sequential CuAAC reactions.

Continuing with their investigations on glycodendrofullerene structures for EBOV infections, Illescas et al. reported the synthesis of a new derivative formed by 13 C_60_ moieties covalently linked and surrounded by 120 mannoses ligands using CuAAC as a central synthetic methodology (Figure 23). Two different spacers between the central C_60_ and the peripheral C_60_ units were used. These new systems exhibited outstanding antiviral activity (IC_50_ in the sub-nanomolar range). These reports showed the importance of the multivalent presentation of carbohydrates taking advance of the 3D structure of C_60_ [139].

Sigwalt et al. reported the construction of [140]. They began using simple malonates to generate C_60_ hexa-adducts, incorporating one azide functionality or twelve terminal alkyne groups. Through the CuAAC coupling between twelve azide-functionalized C_60_ hexa-adducts and a central C_60_ hexa-adduct with twelve terminal alkyne groups, they achieved a yield of up to 83% at room temperature in a DCM/H₂O medium. This approach highlights the ability to efficiently synthesize large, functional macromolecules by incorporating ester groups in simple steps using click reactions. This method enhances the application of C_60_ derivatives as drug-delivery systems by improving their solubility in aqueous media.

Despite the versatility of CuAAC reaction for coupling C_60_ with carbohydrate units, it faces challenges for larger systems, where the chelating ability of these moieties [155,156] and the triazole rings [157] toward copper ions are exacerbated by the crowded environment generated. This chelation hinders complete functionalization, reduces yield, and complicates the removal of cytotoxic copper post-reaction. To address these issues, copper-free click reactions have been employed.

**Figure 24:**
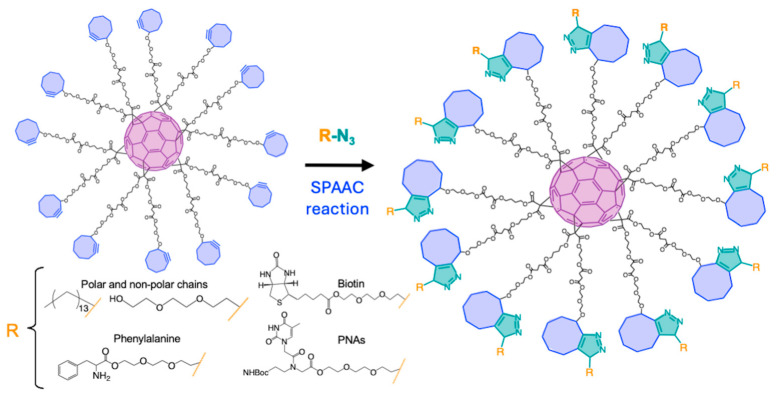
Symmetric C_60_ hexa-adduct substituted with twelve cyclooctyne moieties to carry out SPAAC reactions with different azides appended with polar and non-polar chains, biotin, PNA, and thymine.

In this regard, Ramos-Soriano, et al. [143] synthesized a highly symmetric C_60_ hexa-adduct (4.2 A, Figure 19), appended with 12 cyclooctyne moieties to promote copper-free SPAAC click reactions (Figure 24). This derivative demonstrated versatility to attach polar, non-polar chains and biomolecules like biotin, amino acids such as phenylalanine (PNA), and peptides decorated with nucleic acid monomers like thymine, all of them previously functionalized with azide groups. All compounds were obtained in DMSO under microwave irradiation at 50 °C for 30 min, achieving excellent yields of over 90%. This copper-free methodology is particularly suitable for preparing globular fullerene derivatives for biological applications.

Illescas et al. continued their research on glycodendrofullerenes, synthesized by SPAAC reaction, to target the Zika (ZIKV) and Dengue viruses (DENV). The entry of these pathogens to the host cell takes place through DC-SIGN. The blocking of this receptor through multivalent glycoconjugates supposes a promising biological target to inhibit the infection process. To get enhanced multivalency, they developed a tridecafullerene appended with 120 or 360 1,2-mannobiosides, depending on the used linker, by the coupling of the corresponding alkyne precursors and the azide-functionalized glycofullerenes by heating the reaction at 50 °C under microwave irradiation. The antiviral activity of these compounds was tested for the inhibition of ZIKV and DENV, exhibiting a very strong response at pM concentrations. A compound with 120 saccharide residues showed an IC_50_ of 516 pM for the ZIKV and an IC_50_ of 98 pM for the DENV, whereas a compound with 360 mannobiosides showed the greatest inhibitory activity with an IC_50_ of 67 pM for the ZIKV and an IC_50_ of 35 pM for the DENV [144].

#### 4.1.2. Different Addends (B)

The presence of multiple identical addends is very important for inducing a cluster effect to enhance the affinity toward a biological target, for example, in saccharide-lectin interactions.

On the other hand, the presence of different addends allows the development of synergic interactions or the obtaining of hybrid materials with targeting and therapeutic moieties. Click chemistry plays an important role as many of the developed reactions are orthogonal and permit selective functionalization depending on the reaction conditions or the present substrates.

In this section, we presented the combination of different click chemistry protocols to obtain multiple adducts of C_60_ with different peripheral addends. It is noticed that the most used approaches are CuAAC, SPAAC, thiol–maleimide, and IEDDA.

**Figure 25:**
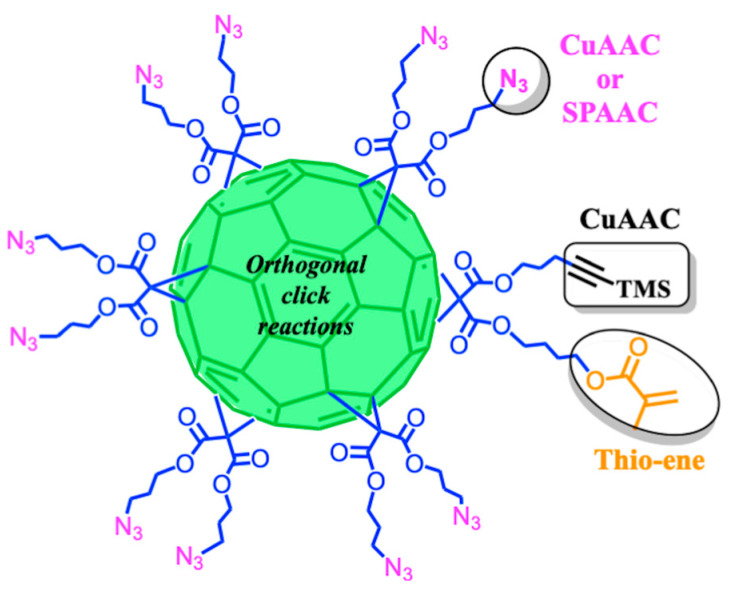
Development of hetero-arm fullerene hexa-adducts for orthogonal functionalization.

In 2010, Iehl and Nierengarten reported a novel hexakis Bingel-Hirshc C_60_ derivative with three different functional moieties for orthogonal click transformations [158]. As a first step, they prepared the mono adduct with a hetero-arm malonate containing a TMS-protected alkyne for CuAAC in one arm, and an acrylate moiety for thiol–ene on the other arm (Figure 25). Then, this mono adduct was functionalized to the hexakis adduct with azide terminal malonates for the posterior application of CuAAC reaction. This system showed the potential of sequential functionalization by using small organic molecules (Figure 25).

**Figure 26:**
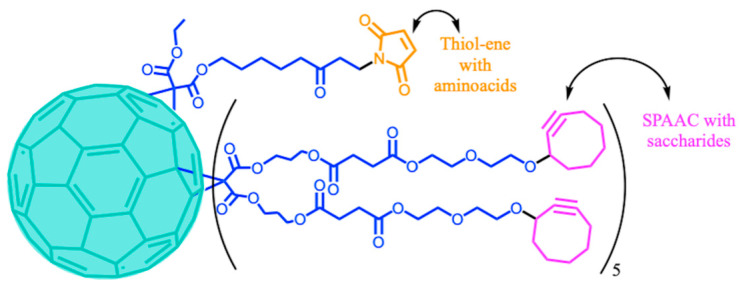
Orthogonal use of thiol–ene and SPAAC click reactions.

Ramos-Soriano et al. [132] described the synthesis of multivalent systems based on C_60_ hexa-adducts using copper-free click reactions like thiol–ene and SPAAC. They synthesized an asymmetric derivative with one maleimide and 10 cyclooctyne units (Figure 26), enabling the simultaneous addition of different addends to C_60_ via thiol–ene and SPAAC click reactions using 1-octanethiol and 2-(2-(2-azidoethoxy)ethoxy)ethan-1-ol, respectively, achieving yields of over 96%, with microwave irradiation at 50 °C for 30 min in DMSO. This strategy enabled the coupling of amino acids and saccharides to C_60_, ensuring the biocompatibility of the new conjugates.

Vitra et al. reported the synthesis of spherical nucleic acids (SNAs) by stoichiometric controlled functionalization of a dodeca-azido fullerene using SPAAC reaction. As a first step, they obtained the monofunctionalized C_60_ after the substoichiometric reaction of DOTA and Alexa 488 oligonucleotide markers, both modified with cyclooctyne, with the azide-functionalized 12-armed C_60_. Subsequently, the product was exposed to an excess of different oligonucleotides to yield the mono-labeled full-armed SNAs. These SNAs are useful as highly selective delivery vehicles because they exhibit efficient cellular uptake through endocytosis, demonstrating muted innate immune responses and resistance to nuclease degradation, avoiding renal clearance due to their sizes [145].

Flos et al. [141] synthesized eight novel C_60_ conjugates containing mono- and disaccharide motifs commonly found in biologically relevant multivalent targeting lectins, using CuAAC reaction. A first set of glycofullerenes were formed with 12 identical addends of either α-D- mannopyranosyl, β-D-galactopyranosyl, α-L-fucopyranosyl or β-lactosyl (4.2 A, Figure 19). A second set containing different addends with one sp^2^-iminosugar (1N-ONJ) glycomimetic motif with a C6 linker and ten of the mentioned glycoside motifs was also synthesized (4.2 B, Figure 19). The products were obtained in yields of up to 88%. The glycofullerenes were tested for the inhibition of enzymes like Jack bean α-mannosidase (JbMan), α-amylase, and β-galactosidase, showing IC_50_ values between 2.2 and 18 µM. The derivative with different addends, including α-D-mannopyranosyl motif and the unit of 1N-ONJ, exhibited the highest potency. This study underscores the potential of multivalent glycosystems in enzyme inhibition for therapeutic applications.

**Figure 27:**
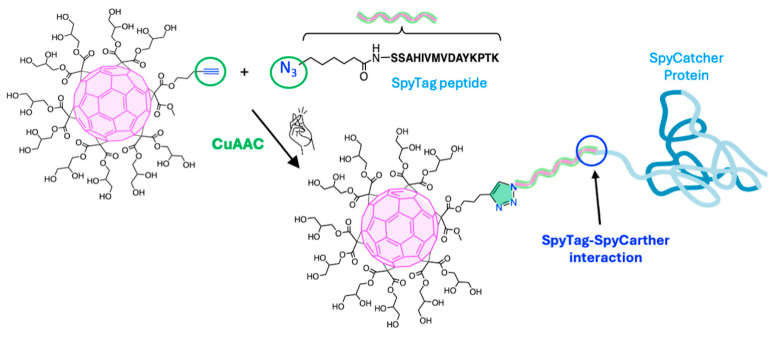
C_60_-protein bioconjugate by CuAAC click reaction and SpyTag-SpyCatcher spontaneous interaction.

Yin et al. [142] reported the synthesis, via CuAAC reaction, of water-soluble C_60_ hexa-adduct bearing the Streptococcus pyogenes surface (SpyTag) unit, a peptide that spontaneously binds to the SpyCatcher protein (4.2 B, Figure 19), which is relevant for further protein linkage. The click reaction between alkyne-functionalized C_60_ and azide end-capped SpyTag enabled effective SpyCatcher binding despite the bulk of the C_60_ derivative (Figure 27). The resultant bioconjugate could modulate specific protein-protein interactions under physiological conditions, and thus may be promising for applications in drug development, particularly those requiring improved cell penetration.

## 5. Conclusions and Final Remarks

Click chemistry has become an essential methodology for developing new pharmacologically active molecules, drug carriers, and biomarkers, and, as noted here, for enhancing the properties of molecular platforms like polymers, cyclodextrins, and fullerenes, for their use in medical applications. While the copper-catalyzed azide–alkyne cycloaddition (CuAAC) reaction was the first developed click approach and continues to be the most widely employed, alternative strategies such as the thiol–ene reaction (a classic Michael addition), the inverse electron-demand Diels–Alder (IEDDA) reaction, and the strain-promoted azide–alkyne cycloaddition (SPAAC) reaction—which does not require copper catalysts—have rapidly gained importance in the pharmaceutical field. Although these types of reactions exhibit numerous advantages including versatile orthogonality, a modular design, simple protocols with high yields, and stereospecificity, some limitations may arise when biomacromolecules are involved. The introduction of clickable groups on entities like proteins or nucleic acids must be carried out by conventional protocols using organic solvents that might affect their integrity and, therefore, their biological function. Additionally, the thiol residues present in some proteins may react as Michael donors, decreasing the orthogonality of reactions like SPAAC or thiol–ene.

From the standpoint of green chemistry, click reactions generate minimal harmful waste with a desirable atomic economy.

This review emphasized the effectiveness of the click strategies for coupling sterically hindered molecules, resulting in the formation of macromolecular entities with considerable potential for applications in nanomedicine. With ongoing advances in this field, we anticipate the development of new materials for treating emergent infectious diseases, cancer, and other health human conditions. Furthermore, the robustness of the click concept permits the incorporation of novel synthetic strategies that will expand the range of attachable specific functional groups for targeted treatment of various diseases.

## Figures and Tables

**Table 1:** Summary of click reactions for functionalization of polymeric systems.

Click Reaction	Application	System Type	Reference
CuAAC	Co-delivery system	2.1 A	[37]
	Controlled release system	2.1 A	[38]
	Controlled release system and tumor-targeting	2.1 A	[39]
	Targeted drug delivery	2.1 A and 2.1 B	[40]
	Co-delivery system and tumor imaging agent	2.1 A	[41]
	Antibody purification to diagnosis	2.1 B	[42]
	Genetic polymer capable of post-functionalization	2.1 C	[43]
	Drug and gene delivery	2.1 C	[44]
SPAAC	Drug delivery and diagnosis	2.1 B	[45]
	Biomarker to infectious diseases diagnostic	2.1 B	[46]
	Drug delivery, molecular diagnostics, and gene regulation	2.1 C	[47]
	Reversible anticoagulant activity	2.1 D	[48]
	Detection of cell surface nucleolin	2.1 D	[49]
	Targeted cancer therapy	2.1 D	[50]
Thiol–ene	Tissue remodeling	2.1 B	[51]
	3D bioprinting	2.1 B	[52]

**Table 2:** Summary of click reactions for functionalization of cyclodextrins.

**Click Reaction**	**Application**	**System Type**	**Reference**
CuAAC	Controlled drug delivery system	3.1 A	[68]
	Controlled drug delivery system	3.1 A	[69]
	Antibody detection, serum analysis, and diagnosis	3.1 C	[70]
	Drug delivery system	3.1 B	[71]
	Drug delivery system	3.1 C	[72]
	Drug delivery system	3.2 D	[73]
	Controlled drug delivery system	3.2 F	[74]
	Controlled drug delivery systems	3.3 H	[75]
	Drug delivery system	3.3 H	[76]
	MRI Contrast agent	3.3 H	[77]
	Drug delivery system	3.3 G	[78]
	Antibiotic therapy	3.3 G	[79]
	Development of therapies for amyloid-related diseases	3.3 G	[80]
	Drug delivery and diagnostics	3.4 J	[81]
	Controlled drug delivery system	3.4 I	[82]
SPAAC	Cell-based therapies	3.4 J	[83]
Thiol–ene	Tissue engineering	3.2 E	[84]
	Controlled drug delivery system	3.2 E	[85]
	Implants or controlled drug delivery systems	3.2 F	[86]
	Controlled drug delivery system	3.3 H	[87]
Diels–Alder	Controlled drug delivery system	3.2 F	[88]

**Table 3:** Summary of click reactions for functionalization of fullerenes.

**Click Reaction**	**Application**	**System Type**	**Reference**
CuAAC	Tumor therapies and delivery system	4.1	[133]
	Bacterial infection inhibitor	4.2A	[134]
	Bacterial infection inhibitor	4.2A	[135]
	Antiviral agent against HIV and EV71	4.2 A	[136]
	Antiviral agent against EBOV-GP	4.2 A	[137]
	Antiviral agent against EBOV-GP	4.2A	[138]
	Antiviral agent against EBOV-GP	4.2A	[139]
	Solubility enhancement	4.2 A	[140]
	Enzyme inhibition	4.2 A and 4.2 B	[141]
	Enhancement cell penetration	4.2 B	[142]
SPAAC	Bioconjugation	4.2 A	[143]
	Antiviral agent against Zika virus	4.2A	[144]
	Bioconjugation	4.2B	[145]
Thiol–ene	Bioconjugation	4.2B	[145]
